# West Nile virus capsid protein inhibits autophagy by AMP-activated protein kinase degradation in neurological disease development

**DOI:** 10.1371/journal.ppat.1008238

**Published:** 2020-01-23

**Authors:** Shintaro Kobayashi, Kentaro Yoshii, Wallaya Phongphaew, Memi Muto, Minato Hirano, Yasuko Orba, Hirofumi Sawa, Hiroaki Kariwa

**Affiliations:** 1 Laboratory of Public Health, Faculty of Veterinary Medicine, Hokkaido University, Kita-ku, Sapporo, Japan; 2 Division of Molecular Pathobiology, Research Center for Zoonosis Control, Hokkaido University, Sapporo, Japan; 3 Global Institution for Collaborative Research and Education (GI-CoRE), Hokkaido University, Sapporo, Japan; 4 Global Virus Network, Baltimore, Maryland, United States of America; National Institute of Allergy and Infectious Diseases, UNITED STATES

## Abstract

West Nile virus (WNV) belongs to the *Flaviviridae* family and has emerged as a significant cause of viral encephalitis in birds and animals including humans. WNV replication directly induces neuronal injury, followed by neuronal cell death. We previously showed that accumulation of ubiquitinated protein aggregates was involved in neuronal cell death in the WNV-infected mouse brain. In this study, we attempted to elucidate the mechanisms of the accumulation of protein aggregates in the WNV-infected cells. To identify the viral factor inducing the accumulation of ubiquitinated proteins, intracellular accumulation of ubiquitinated proteins was examined in the cells expressing the viral protein. Expression of capsid (C) protein induced the accumulation, while mutations at residues L51 and A52 in C protein abrogated the accumulation. Wild-type (WT) or mutant WNV in which mutations were introduced into the residues was inoculated into human neuroblastoma cells. The expression levels of LC3-II, an autophagy-related protein, and AMP-activated protein kinase (AMPK), an autophagy inducer, were reduced in the cells infected with WT WNV, while the reduction was not observed in the cells infected with WNV with the mutations in C protein. Similarly, ubiquitination and degradation of AMPK were only observed in the cells infected with WT WNV. In the cells expressing C protein, AMPK was co-precipitated with C protein and mutations in L51 and A52 reduced the interaction. Although the viral replication was not affected, the accumulation of ubiquitinated proteins in brain and neurological symptoms were attenuated in the mouse inoculated with WNV with the mutations in C protein as compared with that with WT WNV. Taken together, ubiquitination and degradation of AMPK by C protein resulted in the inhibition of autophagy and the accumulation of protein aggregates, which contributes to the development of neurological disease.

## Author summary

The elimination of ubiquitinated protein aggregates from neuronal cells occurs via protein degradation systems such as autophagy or proteasome. Previously, we determined that WNV infection induces the accumulation of ubiquitinated proteins in neuronal cells. However, the detailed mechanisms underlying this accumulation and its association with pathogenicity in the brain remain unclear. Here, we demonstrated that residues L51 and A52 of WNV C protein are responsible for the accumulation of the ubiquitinated proteins. The two C protein residues promoted the interaction between C protein and the autophagy-inducing enzyme AMPK, resulting in ubiquitination and degradation of AMPK and inhibition of autophagy. A mutation in WNV that impaired the AMPK-C protein interaction attenuated the accumulation of ubiquitinated proteins and neurological symptoms in mice. Our findings are the first to describe the regulation of AMPK by a viral protein and represent a step forward in understanding of molecular mechanisms of the neuropathogenesis of WNV infection.

## Introduction

West Nile virus (WNV) is a member of the Japanese encephalitis virus (JEV) serocomplex within the genus *Flavivirus* of the family *Flaviviridae*. The viral genome constitutes a single-stranded positive-sense RNA of approximately 11 kb that encodes a single polyprotein. This polyprotein is cleaved by cellular and viral proteases into three structural proteins, capsid (C), pre-membrane, and envelope (E) proteins, as well as seven nonstructural proteins, NS1, NS2A, NS2B, NS3, NS4A, NS4B, and NS5. In nature, mosquitoes and birds commonly harbor WNV reservoirs, whereas mammals, such as humans or horses, are incidental hosts. Clinical symptoms of WNV infection include febrile illness, fatal meningoencephalitis, and acute flaccid paralysis [1]. Although some vaccines are available for use in animals, no vaccine or specific therapy against WNV is currently approved for human use.

WNV infection of the central nervous system (CNS) induces neuroinflammation and neuronal loss [1]. Neuronal cells are the primary targets of WNV infection, which results in neuronal cell death [1]. Although WNV replication directly induces neuronal cell death, the mechanisms underlying this cell death remain unknown. Previously, we discovered the accumulation of ubiquitinated proteins in neuronal cells infected with WNV in mice [2] and a neurofibrillary tangle, formed by abnormal phosphorylated tau protein, was observed in the brain of a WNV-infected individual with encephalomyelitis [3]. The accumulation of ubiquitinated proteins is the hallmark of neurodegenerative diseases such as Alzheimer’s disease, and the aggregation of ubiquitinated proteins has been associated with neuronal cell death [4]. The accumulation of ubiquitinated proteins was reported as one of the causes of neurodegeneration following human immunodeficiency virus (HIV) infection [5]. Therefore, the accumulation of ubiquitinated proteins is considered important in the pathogenesis of neurological diseases induced by WNV infection; however, the detailed mechanisms underlying the pathogenicity in the brain are not well understood.

Protein aggregation are rarely observed in healthy cells because they are degraded by protein degradation systems that maintain cellular homeostasis [6]. Among these systems, autophagy is closely associated with the elimination of protein aggregates [7]. Autophagy is a catabolic process involved in the turnover of organelles and macromolecules and is induced in response to diverse stimuli, including nutrient starvation, cytokine release, and pathogenic infection [8, 9]. Notably, autophagy impairment is associated with the development of various diseases, including neurodegenerative diseases [10, 11] but the impairment mechanism is not clearly understood.

The autophagic pathway can play an antiviral role through disruption of the replication mechanism of many viruses, including WNV [9, 12]. Several viruses, such as herpes simplex virus type 1 (HSV-1) and HIV type 1, regulate the essential factors for autophagy for viral replication [13, 14]. Thus, autophagy regulation is suggested to influence the pathogenesis of viral infection. Therefore, we hypothesized that WNV regulates protein degradation systems through autophagy inhibition, resulting in the protein aggregation, which further lead to cell death and neurological disease.

In this study, we investigated the mechanism underlying the accumulation of ubiquitinated proteins in neuronal cells infected with WNV. We found that C protein induced the accumulation of ubiquitinated proteins and inhibited autophagy through degradative interactions with autophagy-related host factors. Consequently, these interactions influenced the accumulation of ubiquitinated protein and the pathogenicity of WNV infection.

## Results

### WNV C protein induced accumulation of protein aggregates

**Fig 1:**
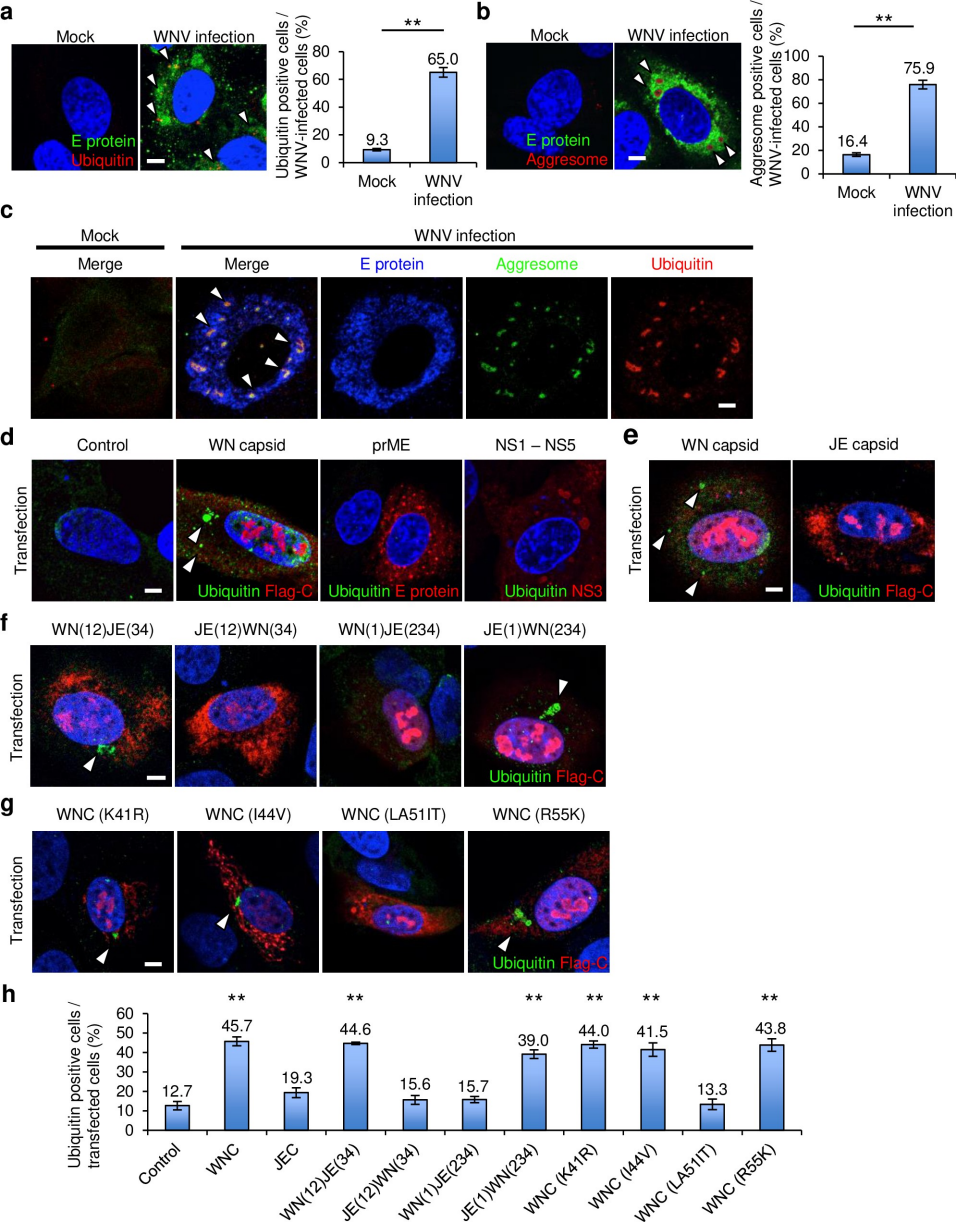
C protein of WNV induced the accumulation of protein aggregates. (a–c) Accumulation of protein aggregates. SK-N-SH cells were infected with mock or WNV [1 pfu/cell]. The cells were harvested at 48 hpi and stained for E protein (green), ubiquitin (a), or aggresome (b) (red), and nuclei (blue). Number of ubiquitin (a) or aggresome (b) -positive cells per WNV-infected cells was counted using the Fiji image software. Data represent the means ± standard error of three independent experiments. Statistical significance was assessed using a two-tailed Student’s t-test. **p < 0.01. (c) Colocalization of ubiquitin (red) and aggresome (green) was observed in the cells positive for E protein (blue). Arrowheads indicate the signals of ubiquitin (a), aggresome (b), or colocalization of ubiquitin and aggresome (c). Scale bars: 5 μm. (d) C protein expression induced ubiquitin accumulation. SK-N-SH cells were transfected with the plasmids expressing mock, flag-C, prME, or all of NS protein and cultured for 48 h. The cells were stained for ubiquitin (green), flag-C, E, or NS3 protein (red), and nuclei (blue). Arrowheads indicate ubiquitin signals. Scale bar: 5 μm. (e–g) Identification of C protein amino acids responsible for ubiquitin accumulation. The cells transfected with the indicated plasmids were stained for ubiquitin (green), flag-C (red), and nuclei (blue). Arrowheads indicate ubiquitin signals. Scale bar: 5 μm. (g) Number of ubiquitin-positive cells per transfected cells was counted using the Fiji image software. Data represent the means ± standard error of three independent experiments. Statistical significance was assessed using a one-way ANOVA (F = 38.43, p = 2.48e-12) followed by Dunnett’s test. **p < 0.01.

We previously demonstrated that ubiquitinated proteins were accumulated in neuronal cells infected with WNV *in vivo* and *in vitro* [2]. Protein aggregates accumulate and form inclusion bodies, termed the aggresome, in neuronal cells in neurodegenerative diseases [15]. The aggresome is detected by a fluorescent molecular rotor dye that binds to the tertiary structure of the aggregated proteins [16, 17]. We examined the accumulation of ubiquitinated proteins and aggresomes in cells infected with WNV. The number of ubiquitin- or aggresome-positive cells was significantly higher in cells infected with WNV than in mock cells (Fig 1A and 1B), and the aggresomes completely co-localized with ubiquitin (Fig 1C), indicating that WNV infection induced the aggregation of ubiquitinated proteins in neuronal cells.

Next, we attempted to identify the viral protein responsible for inducing the accumulation of protein aggregates. SK-N-SH cells were transfected with plasmids expressing C, prME, or entire genes from NS1–NS5, which can function as replication complexes. Ubiquitin accumulation was observed in cells expressing C protein but not in those expressing prME or NS1–NS5 (Fig 1D). These findings imply that the C protein of WNV (WNC) induced the accumulation of protein aggregates.

We also examined the effect of the C protein of JEV (JEC), which belongs to the same JEV serogroup as WNV, on the accumulation of protein aggregates. Ubiquitin accumulation was not observed in cells expressing JEC (Fig 1E), and the number of ubiquitin-positive cells significantly increased in cells expressing WNC compared with mock-treated cells, whereas it did not increase that in cells expressing JEC (Fig 1H).

Both WNC and JEC comprise four alpha-helices (S1A Fig). To identify the region of ubiquitin accumulation, plasmids were constructed in which the helices were interchanged between WNC and JEC (S1A Fig). The number of ubiquitin-positive cells was significantly higher in cells expressing C proteins comprising the second alpha-helix derived from WNV (Fig 1F and 1H). A difference of five amino acids was detected in the second alpha-helix between WNC and JEC (S1A Fig). To identify the critical amino acid for ubiquitin accumulation, plasmids were constructed in which substitutions of the different amino acids were introduced, and ubiquitin accumulation was examined in each of the plasmid-transfected cells. The accumulation was unaffected by amino acid substitutions at positions 41 (K41R), 44 (I44V), or 55 (R55K), whereas accumulation decreased in cells expressing the C protein with substitutions at positions 51 and 52 (LA51IT) (Fig 1G and 1H). Furthermore, JEC with substitutions at positions 51 and 52 (IT51LA) induced the accumulation of ubiquitin (S1B and S1C Fig). The intracellular distribution of WNC or JEC was unaffected by any of the substitutions (Fig 1H and S1B Fig). These results indicate that L51 and A52 in WNC were responsible for the accumulation of protein aggregates.

### Accumulation of protein aggregates by WNV C protein was associated with cell death

**Fig 2:**
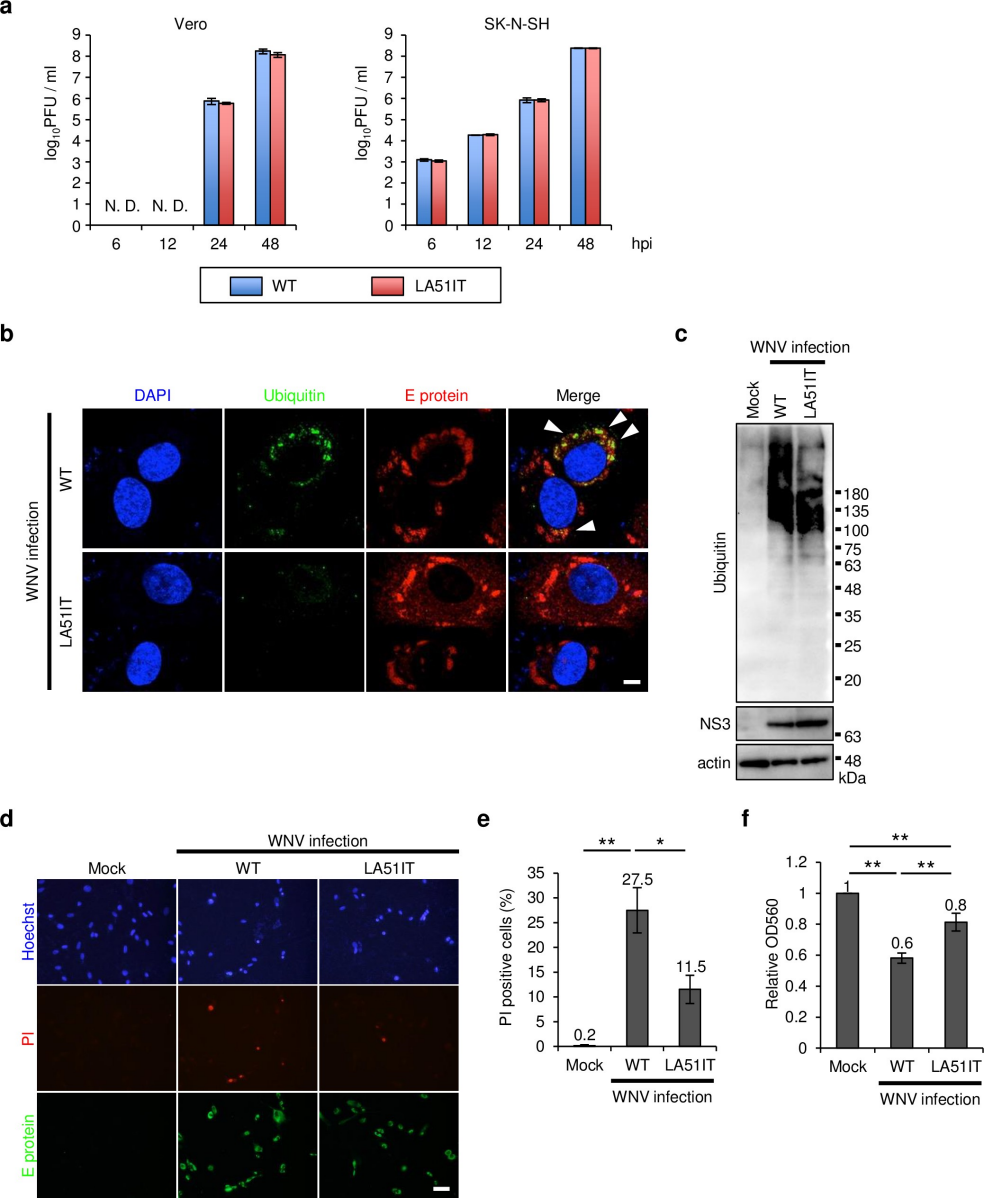
Mutations of L51 and A52 attenuated the accumulation of protein aggregates and cell death. (a) Comparison of viral replication between the WT and LA51IT WNV. Vero cells or SK-N-SH cells were inoculated with WNV WT or LA51IT (0.001 pfu/cell for Vero cells and 1 pfu/cell for SK-N-SH cells), culture supernatants were harvested at 6, 12, 24, or 48 hpi, and viral titers were determined by the plaque assay. Data represent the means ± standard error of three independent experiments. (b and c) The accumulation was reduced by the mutation of L51 and A52. SK-N-SH cells were inoculated with WNV WT or LA51IT (1 pfu/cell) and harvested at 48 hpi. (b) The cells were stained for ubiquitin (green), E protein (red), and nuclei (blue). Arrowheads indicate ubiquitin signals. Scale bar: 5 μm. (c) The harvested lysates were separated into a Triton X-100-soluble fraction for the detection of NS3 and actin and a Triton X-100-insoluble fraction, for detection of ubiquitin, by immunoblotting. (d and e) Cytotoxicity of WNV WT- or LA51IT-infected cells. (d) SK-N-SH cells were inoculated with WNV WT or LA51IT (1 pfu/cell). The cells were harvested at 96 hpi; dead cells were stained by propidium iodide (PI, red) and immunolabeled for E protein (green). Cell nuclei were counterstained with Hoechst 33342 (blue). Scale bar: 50 μm. (e) PI-positive cells were counted using the Fiji image software. Data represent the means ± standard error of three independent experiments. Statistical significance was assessed using a one-way ANOVA (F = 19.60, p = 0.0023) followed by the Scheffe’s F-test. **p < 0.01, *p < 0.05. (f) Viability of WNV WT- or LA51IT-infected cells. SK-N-SH cells were inoculated with WNV WT or LA51IT (1 pfu/cell). After 72 h incubation, MTT assay was performed and the relative OD560 was calculated. Data represent the means ± standard error of three independent experiments. Statistical significance was assessed using one-way ANOVA (F = 49.73, p < 0.001) followed by Scheffe’s F-test. **p < 0.01.

To examine whether the L51 and A52 mutations in C protein affect viral growth and cytotoxicity, Leu-to-Ile and Ala-to-Thr substitutions were introduced at positions 51 and 52 using a previously developed homologous recombination method to produce mutant WNV (LA51IT) [18]. Vero cells or SK-N-SH cells were infected with wild-type (WT) or LA51IT WNV and analyzed for viral replication. No significant difference in viral titers was observed in either cell type at 6, 12, 24, or 48 h post-infection (hpi) (Fig 2A). We also examined the infection rate of WNV WT or LA51IT in SK-N-SH cells. No significant difference was observed in the number of E protein-positive cells between WT and LA51IT WNV at 24, 48, or 72 hpi (S2A Fig). The C protein that binds viral genomic RNA is important for RNA encapsidation and uncoating during viral replication cycles [19, 20]. Thus, we investigated whether these mutations affect the functions of C protein in the virion in terms of the RNA encapsidation and uncoating processes. We transfected 293T cells with plasmids expressing WT or LA51IT C protein, prM, and E protein, and replicon RNA in which the structural protein gene was replaced with a GFP gene; such transfection produces virus-like particles (VLPs) [21]. To examine RNA encapsidation, the amount of replicon RNA packaged in VLPs in the supernatant from 293T cells was analyzed using quantitative RT-PCR. No significant difference was found in the amount of replicon RNA between the expression of WT and LA51IT C proteins (S2B Fig). Due to the lack of structural protein genes in replicon RNA, VLPs are unable to produce progeny particles [12], and thus by monitoring the GFP expression, replication of the uncoated replicon RNA can be monitored [12]. To examine RNA uncoating, SH-SY5Y cells were inoculated with VLPs with WT or LA51IT C protein that were standardized by incorporating replicon RNA. The number of GFP-positive cells was analyzed by flow cytometry. No significant difference in the number of GFP-positive cells was observed between cells inoculated with WT or LA51IT VLPs (S2C Fig). These results indicate that the L51 and A52 mutations in C protein did not affect the growth properties of WNV.

WNV-infected SK-N-SH cells were analyzed for ubiquitin accumulation and cytotoxicity. In contrast to WNV WT-infected cells, accumulation was rarely observed in WNV LA51IT-infected cells (Fig 2B). The Triton X-100-insoluble fraction contains protein aggregates [10, 11]. Triton-insoluble polyubiquitinated proteins were abundant in WNV WT-infected cells compared with those in mock or WNV LA51IT-infected cells (Fig 2C). WNV-infected cells were stained with propidium iodide (PI) to examine cytotoxicity, which was more evident in WNV WT-infected cells than in mock- or WNV LA51IT-infected cells (Fig 2D and 2E). Furthermore, viability of WNV-infected cells was measured by MTT assay. Consistent with the result of PI staining, the viability of cells infected with WT WNV was significantly lower than that with mock or LA51IT WNV (Fig 2F). These results indicate that the L51 and A52 mutations in C protein attenuated the accumulation of protein aggregates and cytotoxicity but did not affect viral growth.

### L51 and A52 of C protein were responsible for autophagy inhibition

Protein aggregates are degraded by protein degradation systems, such as proteasomes or autophagy, to maintain homeostasis [6]. We investigated whether WNV infection inhibits these systems, thereby resulting in the accumulation of protein aggregates. First, to examine the inhibitory effect of WNV infection on proteasomes, proteasome activity was analyzed in SH-SY5Y cells stably expressing ZsProSensor-1 protein, which is specifically degraded by proteasomes [22]. In these cells, proteasome inhibition by MG132 resulted in the accumulation of green fluorescence (S3A Fig). In contrast, accumulation of green fluorescence was not observed in WNV-infected cells (S3A Fig). These observations indicate that proteasome activity was not noticeably affected by WNV infection.

**Fig 3:**
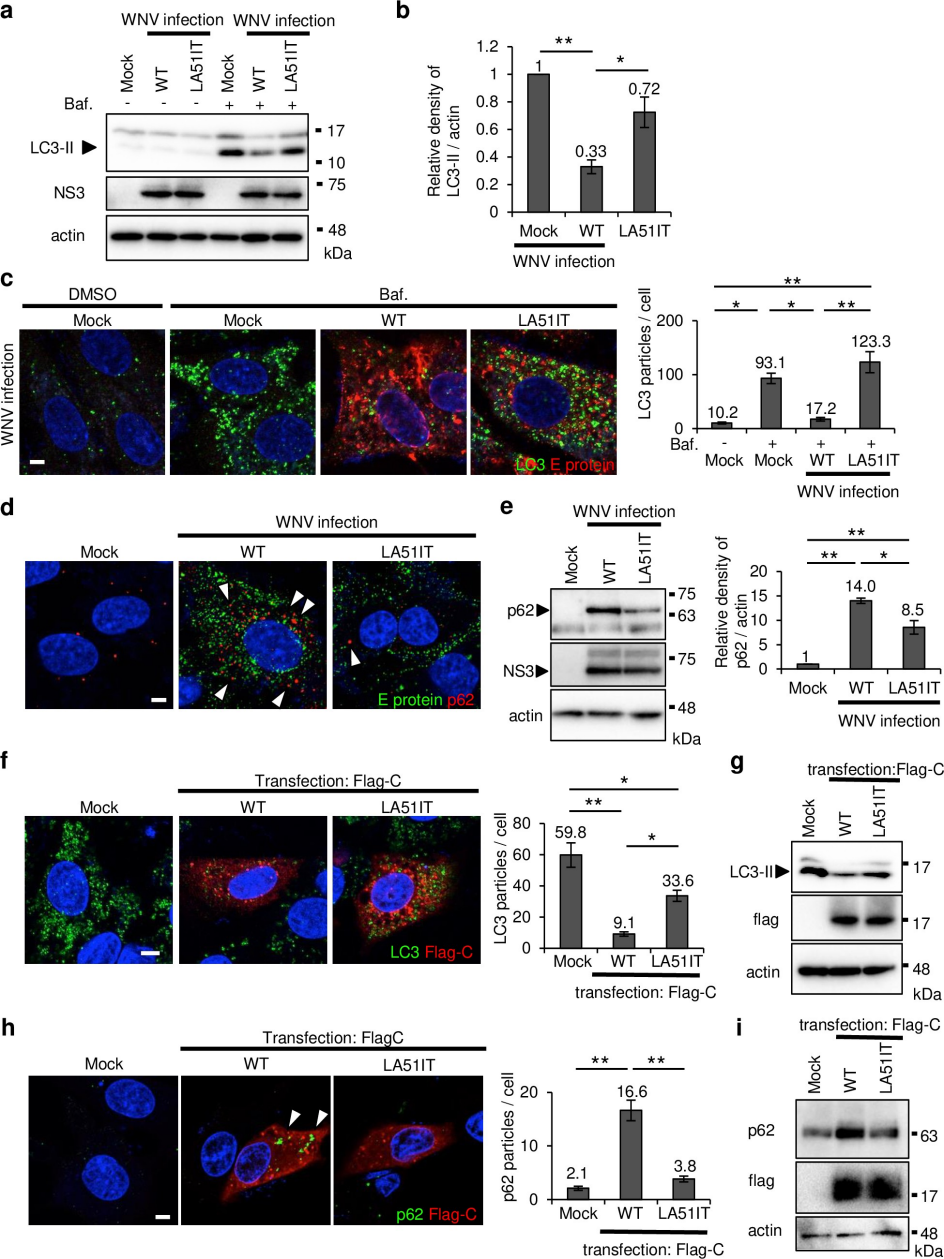
C protein inhibited autophagy. (a–c) WNV infection inhibited autophagosome formation. SK-N-SH cells were infected with WT or mutant LA51IT WNV (1 pfu/cell). After 48 h, the cells were treated with bafilomycin A1 (Baf.) for 6 h and harvested. (a) The cell lysates were analyzed by immunoblotting using anti-LC3, -NS3, and -actin antibodies. (b) The relative band intensity of LC3-II normalized to that of actin was measured. Data represent the means ± standard error of four independent experiments. Statistical significance was assessed using a one-way ANOVA (F = 22.98, p = 0.0003) followed by the Scheffe’s F-test. **p < 0.01, *p < 0.05. (c) The harvested cells were stained for LC3 (green) and E protein (red). Scale bar: 5 μm. The number of LC3 particles per infected cell was counted using the Fiji image software. Data represent the means ± standard error of three independent experiments. Statistical significance was assessed using a one-way ANOVA (F = 12.31, p < 0.001) followed by Scheffe’s F-test. **p < 0.01, *p < 0.05. (d and e) p62 was accumulated by WNV infection. SK-N-SH cells were infected with WNV WT or LA51IT (1 pfu/cell) and harvested at 48 hpi. (d) The harvested cells were stained for E protein (green) and p62 (red). Arrowheads indicate the p62 signals. Cell nuclei were counterstained with DAPI (blue). Scale bar: 5 μm. (e) The cell lysates were analyzed by immunoblotting using anti-p62, -NS3, and -actin antibodies. The relative band intensity of p62 was normalized to that of actin. Data represent the means ± standard error of three independent experiments. Statistical significance was assessed using one-way ANOVA (F = 58.92, p < 0.001) followed by Scheffe’s F-test. **p < 0.01, *p < 0.05. (f-i) C protein expression inhibited autophagy. SK-N-SH cells were transfected with plasmids expressing WT or LA51IT C protein. (f) After 48 h, the cells were treated with Baf. for 6 h and stained for LC3 (green) and flag-C (red). Cell nuclei were counterstained with DAPI (blue). Scale bar: 5 μm. The number of LC3 particles per transfected cell was counted using the Fiji image software. Data represent the means ± standard error of three independent experiments. Statistical significance was assessed using a one-way ANOVA (F = 17.46, p < 0.001) followed by Scheffe’s F-test. **p < 0.01, *p < 0.05. (g) After 48 h, the transfected cells were treated with Baf. for 6 h and were analyzed by immunoblotting using anti-LC3, -flag, and -actin antibodies. (h) The transfected cells were stained for p62 (green) and flag-C (red). Cell nuclei were counterstained with DAPI (blue). Arrowheads indicate p62 immunolabeling. Scale bar: 5 μm. Numbers of p62 particles per transfected cells were counted using the Fiji image software. Data represent the means ± standard error of three independent experiments. Statistical significance was assessed using a one-way ANOVA (F = 52.48, p = 5.52e-14) followed by Scheffe’s F-test. **p < 0.01. (i) The transfected cell lysates were analyzed by immunoblotting using anti-p62, -flag, and -actin antibodies.

Next, we examined the effect of WNV infection on autophagy. To measure autophagosome formation, WNV-infected cells were treated with bafilomycin A1, a lysosomal inhibitor that inhibits the fusion of the autophagosome and lysosome. The level of LC3-II, a marker for autophagosome formation, was significantly reduced in WNV WT-infected cells compared with that in mock cells, and LC3-II reduction was significantly attenuated by the LA mutation (Fig 3A and 3B). WNV WT infection significantly reduced the number of punctate structures of LC3, representing autophagosome formation [23], in cells treated with bafilomycin A1, whereas this reduction was rarely observed in WNV LA51IT-infected cells (Fig 3C and S3B Fig). To confirm that the punctate structures of LC3 were autophagosomes and not aggregated LC3, the punctate structures were examined in Atg5-knockout SH-SY5Y cells (Atg5 KO), in which the expression of both Atg5 and LC3-II was almost completely suppressed (S3C Fig). The punctate structures were hardly observed in Atg5 KO cells treated with bafilomycin A1 (S3D Fig). Furthermore, the accumulation of p62, a protein that is specifically degraded by autophagy, was observed in WNV WT-infected cells, but p62 accumulation was significantly attenuated by the LA mutation (Fig 3D and 3E), whereas mRNA expression of p62 remained nearly the same (S3E Fig). These results indicate that WNV infection inhibited autophagy and that this effect was mediated by C protein via the L51 and A52 residues.

Finally, autophagy activity was examined in cells expressing WT or LA51IT C protein. The number of punctate structures of LC3 in cells expressing WT C protein was significantly reduced compared with that in cells expressing mock protein, and this reduction was significantly attenuated in cells expressing LA51IT C protein (Fig 3F). The level of LC3-II was reduced in cells expressing WT C protein compared with the mock cells, and LC3-II reduction was attenuated by the LA mutation (Fig 3G). The accumulation of p62 in cells expressing WT C protein increased relative to that in the mock-expressing cells, and this upregulation was attenuated in cells expressing LA51IT C protein (Fig 3H and 3I). These results indicate that WNC inhibited autophagy through L51 and A52.

### AMPK expression was regulated through C protein interaction

**Fig 4:**
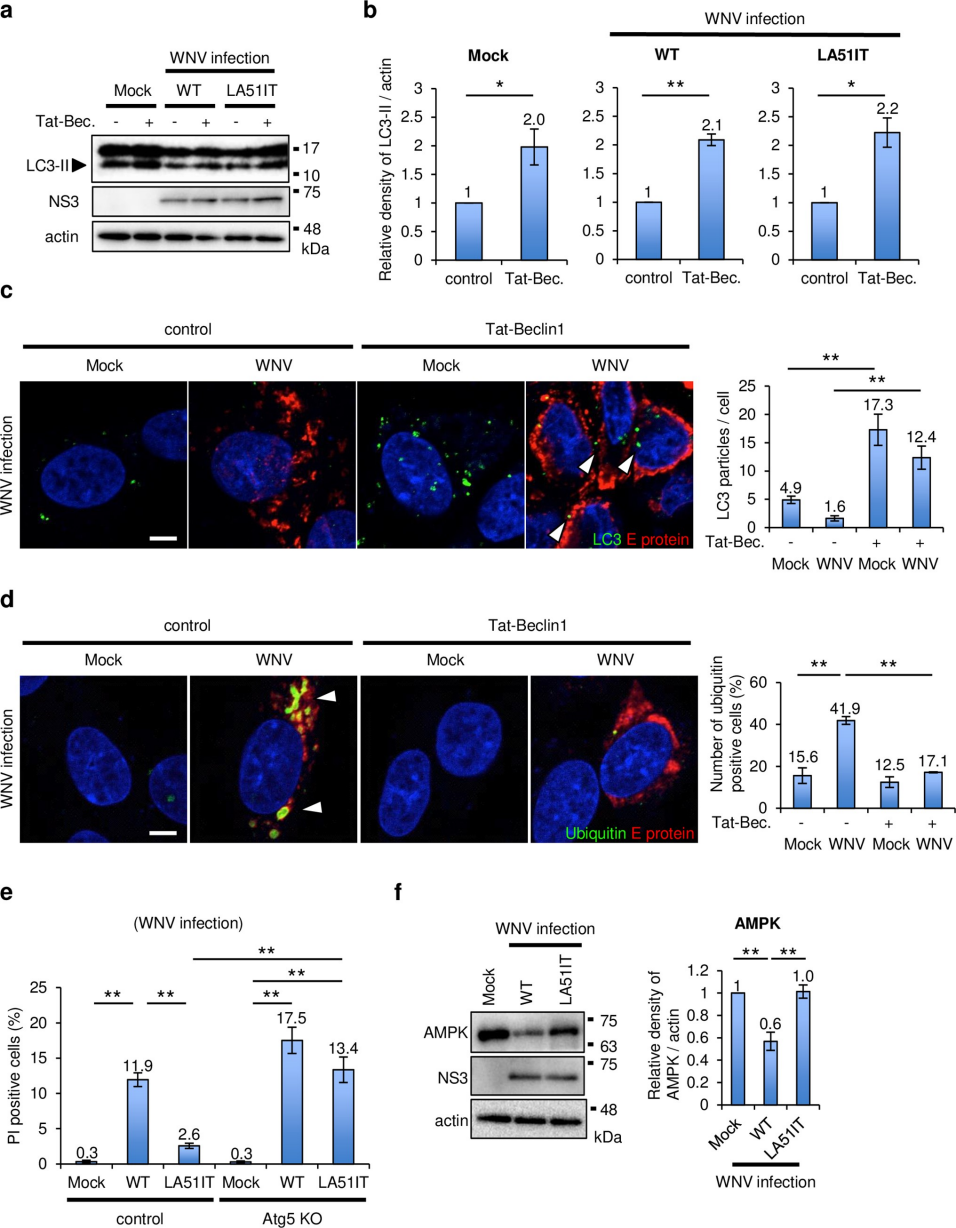
WNV infection inhibited the expression of AMPK and ULK1. (a–d) Autophagy induction in cells treated with Tat-beclin1. SK-N-SH cells were infected with WT or LA51IT WNV (1 pfu/cell). After 48 h, the cells were treated with Tat-beclin1 (Tat-Bec.) for 3 h and harvested. (a) The cell lysates were analyzed by immunoblotting using anti-LC3, -NS3 and -actin antibodies. (b) The relative band intensity of LC3-II normalized to that of actin was measured. Data represent the means ± standard error of three independent experiments. Statistical significance was assessed using a two-tailed Student’s t-test. **p < 0.01, *p < 0.05. (c) The harvested cells were stained for E protein (red) and LC3 (green). Cell nuclei were counterstained with DAPI (blue). Arrowheads indicate LC3 signals. Scale bar: 5 μm. Numbers of LC3 particles per WNV-infected cells were counted using the Fiji image software. Data represent the means ± standard error of four independent experiments. Statistical significance was assessed using a one-way ANOVA (F = 15.80, p < 0.001) followed by the Tukey-Kramer test. **p < 0.01. (d) The harvested cells were stained for E protein (red) and ubiquitin (green). Cell nuclei were counterstained with DAPI (blue). Arrowheads indicate ubiquitin signals. Scale bar: 5 μm. Number of ubiquitin-positive cells per transfected cells was counted using the Fiji image software. Data represent the means ± standard error of three independent experiments. Statistical significance was assessed using a one-way ANOVA (F = 30.87, p < 0.001) followed by Tukey–Kramer test. **p < 0.01. (e) Protective effect of autophagy on WNV-induced cell death. SH-SY5Y Cas9 cells (control) or SH-SY5Y Atg5 KO cells (Atg5 KO) were infected with WT or LA51IT WNV (1 pfu/cell). The cells were harvested at 48 hpi, and dead cells were stained with PI. PI-positive cells were counted using the Fiji image software. Data represent the means ± standard error of three independent experiments. Statistical significance was assessed using a one-way ANOVA (F = 26.50, p < 0.001) followed by Scheffe’s F-test. **p < 0.01. (f) Inhibition of AMPK in the cells infected with WNV. SK-N-SH cells were infected with WNV WT or LA51IT (1 pfu/cell) for 48 h. The cell lysates were analyzed by immunoblotting using anti-AMPK, -NS3, and -actin antibodies. The relative band intensity of AMPK was normalized to that of actin. Data represent the means ± standard error of four independent experiments. Statistical significance was assessed using a one-way ANOVA (F = 19.21, p = 0.0025) followed by Scheffe’s F-test. **p < 0.01.

Autophagosome formation is regulated by AMPK and Beclin1-PI3KC3 complexes (S4A Fig) [24]. We hypothesized that WNV infection influences the factors related to autophagosome formation. To clarify the mechanism underlying autophagy inhibition by WNV infection, WNV-infected SK-N-SH cells were treated with Tat-beclin1, an autophagy-inducing peptide [25], and LC3 expression was analyzed. LC3-II expression was induced by Tat-beclin1 in WNV WT-infected cells, and was similar to expression levels in mock- or WNV LA51IT-infected cells (Fig 4A and 4B). Tat-beclin1 treatment significantly induced punctate structures of LC3 in cells infected with WNV WT or in cells expressing C protein (Fig 4C and S4B Fig). Furthermore, ubiquitin accumulation by WNV infection or by C protein expression was significantly inhibited in cells treated with Tat-beclin1 (Fig 4D and S4B Fig). The number of PI-positive cells significantly decreased in WNV-infected cells following treatment with Tat-beclin1 (S4C Fig). These results suggest that WNV infection had little effect on the regulatory role of PI3KC3 in autophagy induction, and that induction of autophagy decreased the accumulation of protein aggregates and inhibited cell death by WNV infection.

To examine the effect of autophagy on cell death by WNV infection, Atg5 KO cells were infected with WNV and stained with PI. In the control cells, the number of PI-positive cells significantly increased after WNV WT infection relative to mock infection or WNV LA51IT infection (Fig 4E). However, in Atg5 KO cells, the number of PI-positive cells was significantly increased by WNV WT and LA51IT infection compared with mock infection, whereas no significant difference was observed between WNV WT and LA51IT infections (Fig 4E). Furthermore, the accumulation of protein aggregates in Atg5 KO cells infected with WT or LA51IT WNV was examined by immunoblotting. In control cells, Triton X-100-insoluble polyubiquitinated proteins were abundant in the WT WNV-infected cells as compared with those in the LA51IT WNV-infected cells, while the accumulation by LA51IT WNV infection was almost same as WT WNV infection in Atg5 KO cells (S4D Fig). Collectively, these data indicate that autophagy-inhibiting WT WNV infection promoted cell death even in those cells in which autophagy functioned normally; however, WNV LA51IT infection, in which attenuated the inhibitory effect on autophagy, promoted cell death only in autophagy-deficient cells.

Next, we examined the expression of AMPK upstream of the PI3KC3 complex in the autophagy induction pathway in WNV. AMPK expression was significantly reduced in WNV WT-infected cells compared with those in mock cells, and the LA mutation attenuated this reduction (Fig 4F). These observations suggest that the AMPK protein levels was decreased by WNV infection through L51 and A52.

**Fig 5:**
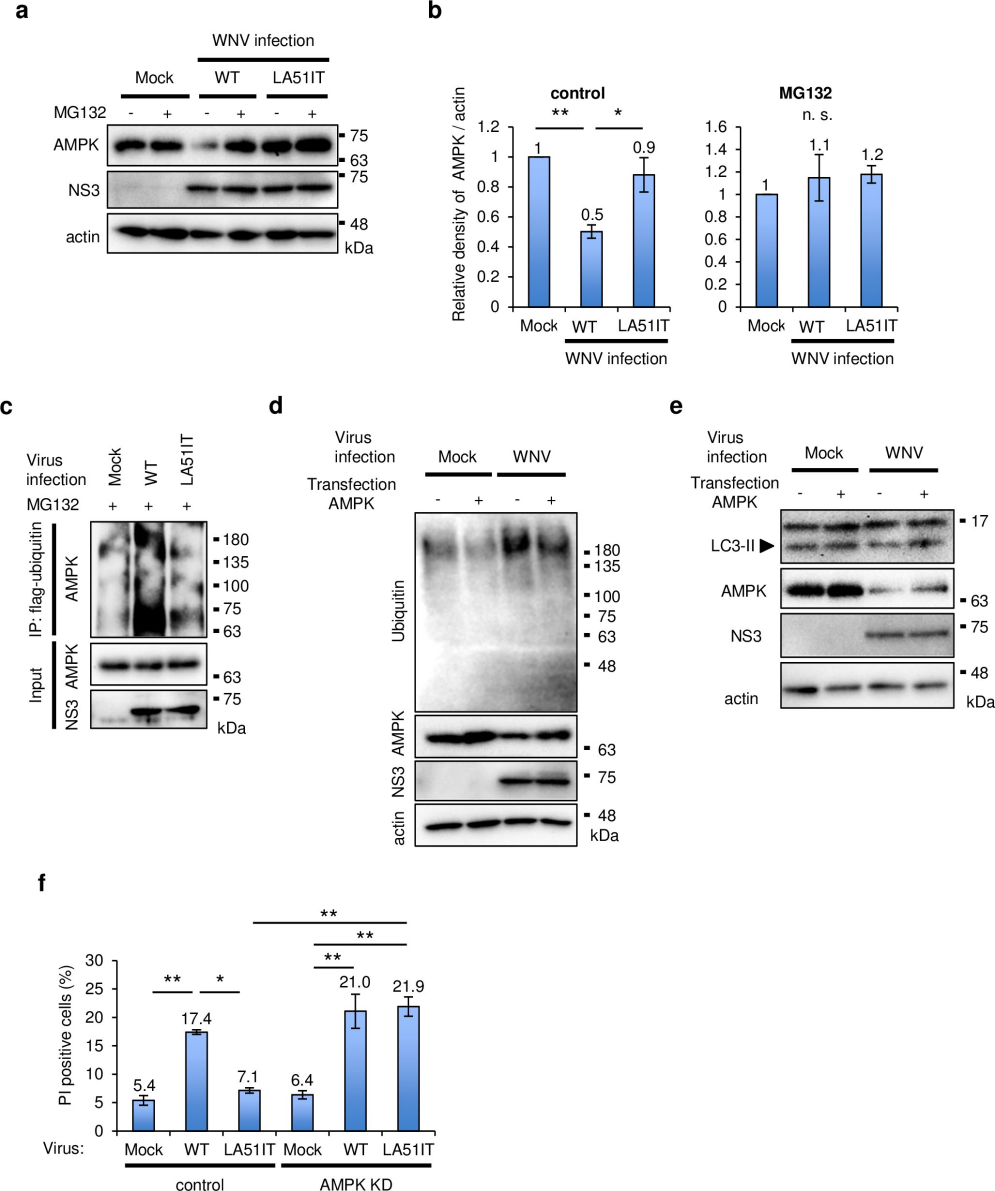
AMPK was degraded by proteasomes in WNV-infected cells. (a and b) AMPK expression was increased by MG132 treatment. SK-N-SH cells were infected with WT or LA51IT WNV (1 pfu/cell). After 36 h, the cells were treated with MG132 for 12 h and harvested. (a) The cell lysates were analyzed by immunoblotting using anti-AMPK, -NS3, and -actin antibodies. (b) The relative band intensity of AMPK normalized to that of actin was measured. Data represent the means ± standard error of four independent experiments. Statistical significance was assessed using a one-way ANOVA (control: F = 13.45, p = 0.002; MG132: F = 0.56, p = 0.59) followed by the Scheffe’s F-test. **p < 0.01, *p < 0.05. (c) AMPK ubiquitination by WNV infection. SH-SY5Y cells were transfected with plasmids expressing flag-ubiquitin and cultured for 24 h. The cells were infected with WNV WT or LA51IT (1 pfu/cell). After 48 h, the cells were treated with MG132 for 3 h before anti-Flag immunoprecipitation (IP) and immunoblotting were performed. (d and e) Effects of AMPK overexpression on the accumulation of ubiquitinated proteins and autophagy. SH-SY5Y cells (d) or SK-N-SH cells (e) were transfected with plasmids expressing AMPK and cultured for 24 h. The cells were infected with WNV WT or LA51IT (5 pfu/cell). After 48 h, the cell lysates were separated into a Triton X-100-soluble fraction for the detection of LC3, AMPK, NS3, and actin and into a Triton X-100-insoluble fraction for the detection of ubiquitin using immunoblotting. (f) Importance of AMPK in WNV-induced cell death. 293T cells (control) or 293T AMPK knockdown cells (AMPK KD) were infected with WT or LA51IT WNV (1 pfu/cell). The cells were harvested at 72 hpi and dead cells were stained with PI. PI-positive cells were counted using the Fiji image software. Data represent the means ± standard error of three independent experiments. Statistical significance was assessed using a one-way ANOVA (F = 26.98, p < 0.001) followed by Scheffe’s F-test. **p < 0.01.

Because AMPK is degraded by the ubiquitin-proteasome system in tumor cells [26], we examined whether AMPK was degraded by this system in WNV-infected cells. The reduction of AMPK expression by WNV WT infection was recovered by the addition of MG132 (Fig 5A and 5B). Furthermore, the expression of AMPK was examined in cells expressing WT, LA51IT, or JEV C protein, which has Ile and Thr at positions 51 and 52. The expression of AMPK was reduced in cells expressing WT C protein but not in those expressing LA51IT or JEV C protein (S5A Fig). Hardly any reduction in AMPK expression was observed in cells treated with MG132 (S5A Fig). NS proteins including NS4A are reported to modulate AMPK signaling in Dengue virus infection [27]. However, the expression of NS4A or all of NS proteins of WNV did not affect AMPK expression (S5B Fig). We also examined the ubiquitination of AMPK in cells infected with WNV. The ubiquitinated proteins were precipitated and AMPK was detected by immunoblotting in the precipitated complex. In cells treated with MG132, WNV WT infection increased the ubiquitination of AMPK compared with that in mock cells, and the ubiquitination of AMPK was decreased by the LA mutation (Fig 5C). On the other hand, there was no noticeable increase in the ubiquitination of AMPK in cells without MG132 treatment (S5C Fig). These results suggest that the ubiquitination and degradation of AMPK were upregulated by WNV infection through L51 and A52.

We investigated whether AMPK regulates the accumulation of protein aggregates and autophagy in WNV-infected cells. The expression of AMPK was decreased by WNV infection and slightly increased by overexpression of AMPK (Fig 5D and 5E). WNV infection induced the accumulation of ubiquitinated proteins in the Triton X-100-insoluble fraction, and this accumulation was reduced in cells overexpressing AMPK compared with that in mock cells (Fig 5D). Overexpression of AMPK compensated for the decrease in LC3-II expression induced by WNV infection (Fig 5E). These results suggest that AMPK is important for the elimination of protein aggregates and the induction of autophagy. To examine the relationship between AMPK degradation and cell death, AMPK-knockdown (KD) cells were produced (S5D Fig) and infected with WNV. In AMPK-KD cells, the number of PI-positive cells significantly increased following WNV WT and LA51IT infection compared with that in mock infection, and the positive rate significantly increased in LA51IT WNV-infected AMPK-KD cells compared with control cells infected with LA51IT WNV (Fig 5F). These results indicate that AMPK degradation was important for the induction of cell death by WNV infection.

**Fig 6:**
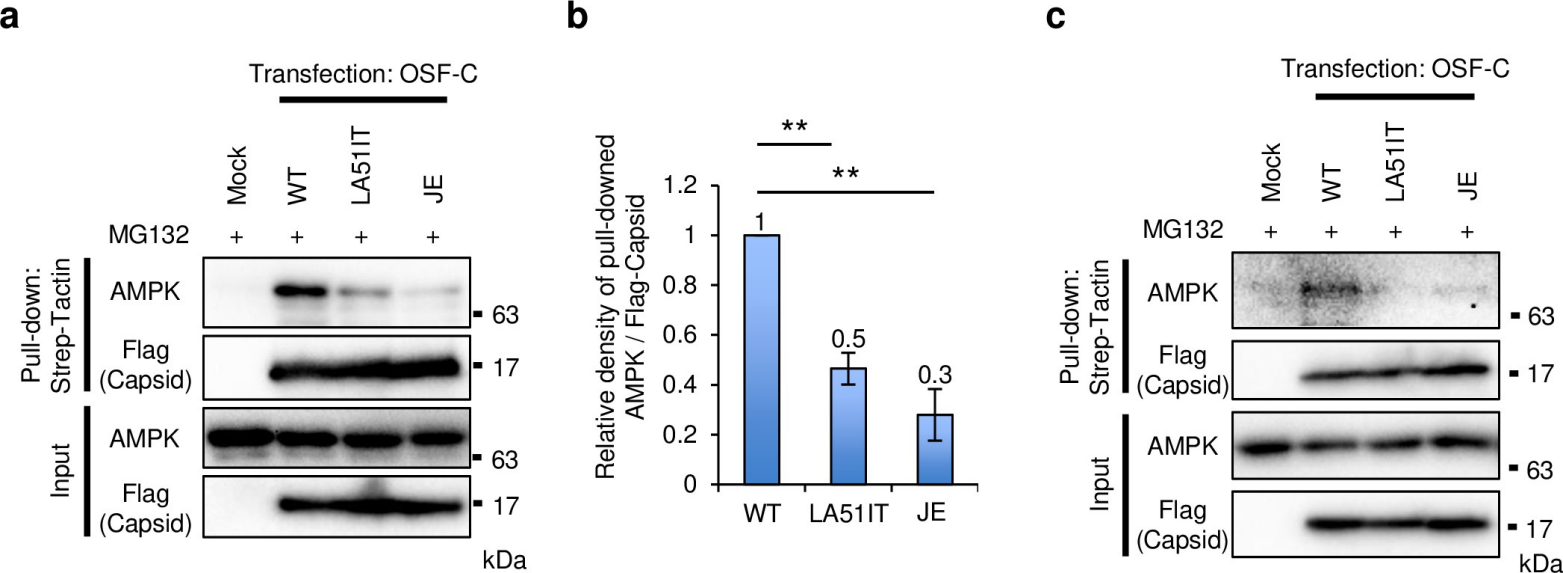
C protein interacted with AMPK. (a and b) Overexpressed AMPK interacted with C protein. SH-SY5Y cells were co-transfected with plasmids expressing AMPK and WT, LA51IT, or JE C protein with One-STrEP-Flag (OSF) tag and cultured for 48 h. The cells were treated with MG132 for 3 h before precipitation by Strep-Tactin. The OSF-C protein complex was analyzed by immunoblotting (a). The relative band intensity of precipitated AMPK normalized to that of precipitated OSF-C protein was measured (b). Data represent the means ± standard error of four independent experiments. Statistical significance was assessed using a one-way ANOVA (F = 28.21, p < 0.001) followed by the Scheffe’s F-test. **p < 0.01. (c) Endogenous AMPK interacted with C protein. SH-SY5Y cells were transfected with the plasmids expressing WT, LA51IT, or JE C protein with OSF-tag and cultured for 48 h. The cells were treated with MG132 for 12 h before precipitation by Strep-Tactin. The OSF-C protein complex was analysed by immunoblotting.

Because AMPK degradation was affected by C protein mutations, we tested whether expressed C protein with One-STrEP-Flag tag interacts with AMPK. Overexpressed AMPK was pulled down with expressed C protein using a Strep-tag in cells treated with or without MG132 (Fig 6A and S5E Fig). The amount of interacted-AMPK in cells expressing LA51IT or JEV C protein was significantly lower than in cells expressing WT C protein (Fig 6B). Furthermore, the interaction between WT C protein and endogenous AMPK was observed, and this interaction was reduced by the LA mutation (Fig 6C). These results indicate that C protein interacted with AMPK through L51 and A52.

### Mutations impeding the accumulation of ubiquitinated proteins attenuated the neuropathogenicity of WNV infection

**Fig 7:**
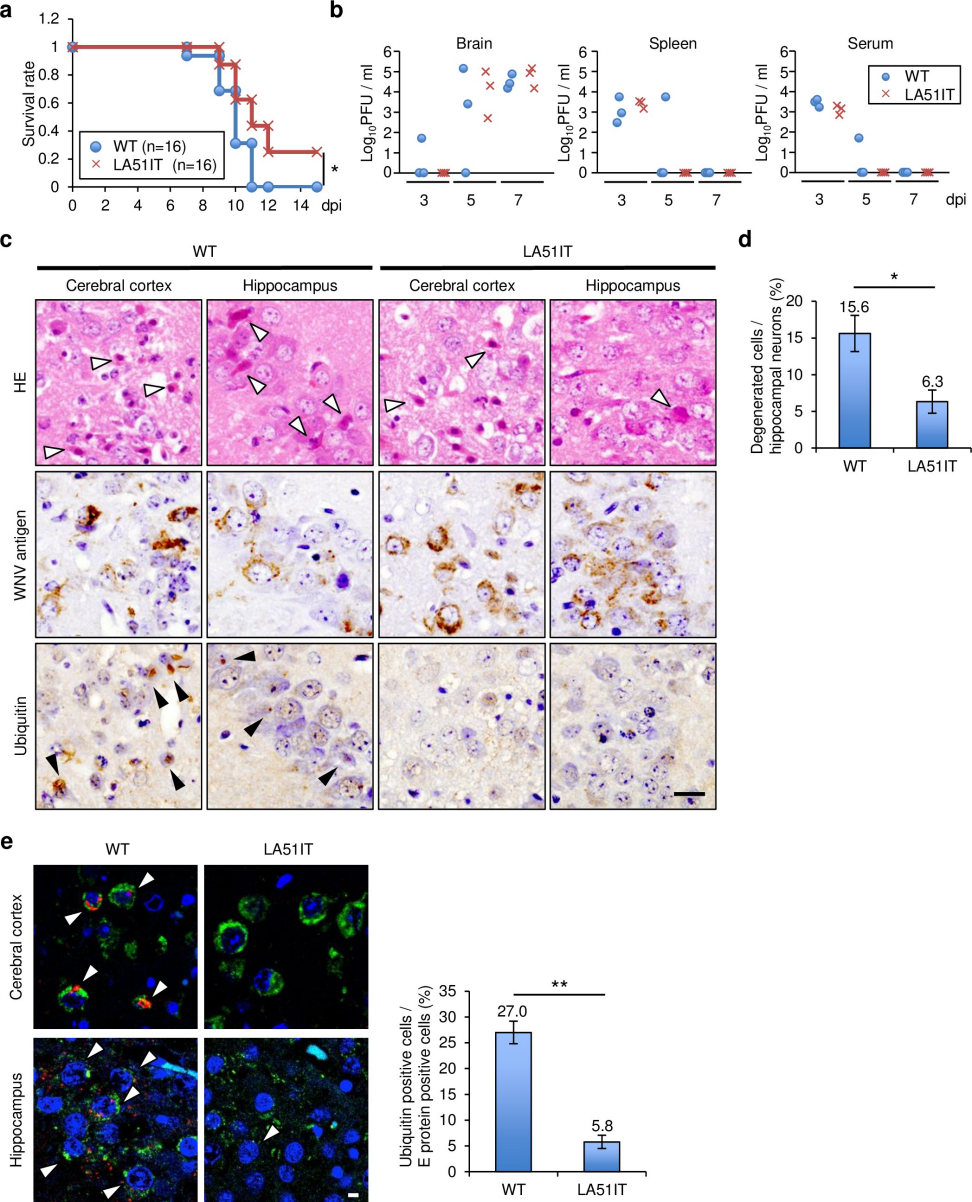
Pathogenicity of LA51IT WNV in a mouse model. Six-week-old female C57BL/6JJmsSlc mice were inoculated with 100 pfu of WT or LA51IT WNV intraperitoneally (a and b) or intracranially (c–e). (a) The Kaplan–Meier survival curves of the mice inoculated with WT or LA51IT WNV. *p < 0.05. (b) The brain, spleen, and serum were collected at 3, 5, or 7 days post infection (dpi) (n = 3), and the viral titers were measured. (c) Cerebral cortical and hippocampal hematoxylin and eosin-stained sections, or sections immunostained for WNV antigen or ubiquitin, from mice inoculated with WNV WT (n = 4) or LA51IT (n = 4). White arrowheads indicate degenerated neuronal cells. Black arrowheads indicate ubiquitin-positive cells. Scale bar: 20 μm. Scale bar: 20 μm. (d) Number of degenerated cells in the CA2 and CA3 hippocampal neuronal layers was counted. Data represent the means ± standard error of four independent experiments. Statistical significance was assessed using a two-tailed Student’s t-test. *p < 0.05. (e) Ubiquitin accumulation in the brain. Sections of cerebral cortex or hippocampus of the mice inoculated with WNV WT or LA51IT were stained with antibodies against ubiquitin (red) and WNV antigen (green). Nuclei were stained with DAPI. White arrowheads indicate ubiquitin-positive cells. Scale bar: 5 μm. The number of ubiquitin-positive cells per E protein-positive cells was calculated. Data represent the means ± standard error of four independent experiments. Statistical significance was assessed using a two-tailed Student’s t-test. **p < 0.01.

C57BL/6JJmsSlc mice were inoculated intraperitoneally or intracranially with WNV WT or LA51IT to evaluate the involvement of the accumulation of ubiquitinated proteins by AMPK degradation and autophagy inhibition during WNV infection in mice. The survival rate after intraperitoneal inoculation significantly increased in mice inoculated with LA51IT compared with that in mice inoculated with WNV WT (Fig 7A). No significant differences in virus titers were observed in the brain, spleen, or serum between the two groups at 3, 5, or 7 days post-infection (dpi) (Fig 7B). After intracranial inoculation, neuronal degeneration with intense eosinophilia of the cytoplasm was observed in many regions of the brain in mice inoculated with WNV WT or LA51IT (Fig 7C). Significantly more degenerated cells in the CA2 and CA3 regions of the hippocampus were present in mice inoculated with WNV WT relative to those with WNV LA51IT (Fig 7D). Ubiquitin was detected in the brains of mice infected with WT, but it was rarely detected in the brains of mice infected with LA51IT (Fig 7C). Consistent with our *in vitro* findings, the number of ubiquitin-positive cells significantly increased in WNV WT-infected cells compared with WNV LA51IT-infected cells (Fig 7E). These findings suggest that the accumulation of protein aggregates contributed to the pathogenesis of WNV infection.

## Discussion

In this study, we investigated the mechanism underlying the accumulation of protein aggregates in neuronal cells infected with WNV and demonstrated that C protein induced AMPK degradation and inhibited autophagy. Moreover, we demonstrated for the first time that the accumulation of protein aggregates may be associated with the pathogenesis of West Nile encephalitis *in vivo*, and proposed a model for cell death in West Nile encephalitis (S6 Fig). C protein interacts with AMPK and mediates the attachment of polyubiquitin to AMPK, and the ubiquitinated AMPK is consequently degraded via the ubiquitin-proteasome system. AMPK degradation results in autophagy inhibition and induces the accumulation of protein aggregates in cells. Such an accumulation of protein aggregates may result in neuronal cell death, leading to the development of neurological disease.

C protein interacts with AMPK and promotes AMPK degradation in the neuropathogenicity of WNV. AMPK is a cellular energy sensor and is activated during metabolic stress [28]. AMPK dysfunction has been suggested to be associated with many diseases [29]. A loss-of-function mutation in AMPK is responsible for Wolff-Parkinson-White syndrome, a congenital condition involving abnormal conductive cardiac tissue [30]. Regarding tumorigenesis, the cancer-specific ubiquitin ligase MAGE-A3/6-TRIM28 and the prolyl isomerase Pin1 participate in the ubiquitination and degradation of AMPK [26, 31]. In this study, we demonstrated that AMPK degradation is associated with neuronal cell death in WNV infection, suggesting that AMPK is critical to the functional maintenance and survival of neuronal cells.

Our study provides evidence of the mechanism underlying the accumulation of protein aggregates in cells infected with WNV. Other studies have demonstrated that conditional deletion of the essential genes for autophagy in the CNS results in the accumulation of ubiquitinated protein aggregates, leading to neurodegeneration and motor deficits in mice [10, 11]. Several viral proteins exhibit anti-autophagic properties that are required for viral replication [32, 33]. HSV-1-encoded neurovirulence protein ICP34.5, viral Bcl 2 protein of Kaposi sarcoma associated herpesvirus, TRS1 and IRS1 proteins of human cytomegalovirus, and HIV-1 protein Nef bind to Beclin1 to inhibit its autophagosome regulatory function [13, 14, 34, 35]. We found that introducing a mutation in C protein that reduced the interaction with AMPK attenuated the inhibition of autophagy and the accumulation of protein aggregates both *in vitro* and *in vivo*. Thus, autophagy inhibition associated with AMPK-degradation is responsible for the accumulation of protein aggregates in neuronal cells infected with WNV. Many factors, including inflammatory responses, affect the pathology of West Nile encephalitis [36]. In addition to those reported conventionally, our results show the importance of neuronal cell death induced by C protein in the pathogenesis of encephalitis.

The accumulation of protein aggregates in neuronal cells is considered to contribute to the development of neuronal disease. However, WNV infection causes acute disease, whereas the progress of many known neurodegenerative diseases, such as Alzheimer’s disease, is relatively slow. The level of ubiquitinated protein aggregates gradually increases during the clinical course of neurodegenerative diseases [4]. In contrast, WNV proteins are abundant and rapidly produced in virus-infected neuronal cells, which may facilitate the rapid accumulation of protein aggregates. These contrasting kinetics of the accumulation of protein aggregates in neuronal cells may be associated with the different neuropathologies of WNV infection and neurodegenerative diseases. Furthermore, a synergistic effect between viral replication and the accumulation of protein aggregates may accelerate the development of acute neuronal degeneration.

Our previous studies demonstrated that autophagy decreased WNV replication, whereas ubiquitinated proteins accumulated in neuronal cells infected with WNV [2, 12]. In other flavivirus infections, suppression of autophagy mediated by E3 ligases such as Nedd4 facilitated the replication of JEV [37]. In addition, activated AMPK, which is an autophagy inducer, inhibited the replication of WNV, Zika virus, and Dengue virus [27, 38]. These results indicate that AMPK-mediated autophagy inhibits flavivirus replication. In this study, autophagy suppressed cell death after infection with WNV LA51IT, which was unable to inhibit autophagy, suggesting that autophagy exerts a protective effect on cell death induced by WNV infection. However, infection of cells with WNV WT (which were competent for inhibiting autophagy) led to cell death even in those cells in which autophagy was normal. Thus, it appears that WNV infection and autophagy are antagonistic, and the inhibition of autophagy by C protein can override the protective effect of autophagy, inducing cell death and neurological disease.

WT C protein was shown to interact with AMPK, and this interaction was significantly decreased by LA mutation. Furthermore, AMPK was ubiquitinated by WNV WT infection, but not WNV LA51IT infection. These results suggest that C protein in the complex with AMPK facilitated the ubiquitination and degradation of AMPK. The proteins degraded by the ubiquitin-proteasome system are labeled with polyubiquitin by E3 ligases. During WNV replication, C protein has been shown to interact with E3 ligases, such as HDM2 [39]. Furthermore, C protein degrades its binding proteins through the proteasome pathway [40]. AMPK has been shown to be ubiquitinated by E3 ligase, recruited by MAGE-A3/6 through its interaction with AMPK, leading to AMPK degradation [26]. Taken together, the results of this and previous studies suggest that C protein is an important mediator of AMPK ubiquitination by certain E3 ligases. Analysis of the C protein-AMPK protein complex may elucidate the mechanisms underlying AMPK degradation in the pathogenesis of WNV infection.

L51 and A52 in C protein are located on the second alpha-helix, a domain associated with viral growth [41, 42] that can structurally interact with lipid bilayers [42]. L51 and A52 are maintained in different strains of WNV but not in most of other neurotropic flaviviruses, such as JEV or tick-borne encephalitis virus (S7 Fig), and these three neurotropic flaviviruses reportedly exhibit different pathogeneses [43]. Several JEV strains encoding L51 and A52 were reported to show higher neuropathogenicity than other strains in a mouse model [44, 45]. These findings suggest that the interaction between AMPK and C protein through L51 and A52 may be important pathogenic factor for Flavivirus infection.

In summary, our findings are the first to describe the regulation of AMPK by a viral protein and the molecular mechanisms through which C protein mediates the neuropathogenesis of WNV. The discovery of this interaction will further improve our understanding of the molecular mechanisms underlying the pathogenicity of WNV infection, and will aid in identifying potential therapeutic pharmacological agonists of AMPK in West Nile encephalitis.

## Materials and methods

### Cells and viruses

SK-N-SH (neuroblastoma) cells, obtained from RIKEN BRC Cell Bank (RCB0426), and Vero cells, obtained from JCRB Cell Bank (JCRB0111), were cultured in MEM (Wako, Osaka, Japan) supplemented with 10% heat-inactivated fetal bovine serum (FBS). SH-SY5Y cells, obtained from ECACC (94030304), were grown in DMEM/Nutrient Mixture F-12 Ham (Wako) supplemented with 10% heat-inactivated FBS. HEK-293T cells, kindly provided by Dr. Matsuura (Osaka University), were cultured in high-glucose DMEM (Wako) supplemented with 10% heat-inactivated FBS. JEV strain JaGAr-01 (Accession number: AF069079) was cloned into plasmids as described below. The WNV 6-LP strain was established from the WNV NY99-6922 strain isolated in 1999 from mosquitoes by plaque purification [46]. All WNV experiments were performed at the Biosafety Level-3 (BSL-3) facility of Hokkaido University, Japan, according to the institutional guidelines.

### Plasmid construction

C protein coding region of WNV or JEV was cloned into a pCXSN-FLAG plasmid, which was generated from the pCMV-Myc plasmid (Takara Bio USA, Inc., Mountain View, CA, USA) by replacing the sequence of the myc tag with that of the FLAG tag and multicloning sequences containing sites for the restriction enzymes *Xho*I, *Sal*I, and *Not*I [47, 48]. The resultant plasmids were labeled pCXSN-fWNC and pCXSN-fJEC, respectively. Plasmids encoding the 1^st^ and 2^nd^ helixes of WNC and the 3^rd^ and 4^th^ helixes of the JEC [WN(12)JE(34)]; the 1^st^ and 2^nd^ helixes of JEC and the 3^rd^ and 4^th^ helixes of WNC [JE(12)WN(34)]; the 1^st^ helix of JEC and the 2^nd^, 3^rd^, and 4^th^ helixes of WNC [JE(1)WN(234)]; or the 1^st^ helix of WNC and the 2^nd^, 3^rd^, and 4^th^ helixes of JEC [WN(1)JE(234)] were produced utilizing the In-Fusion HD Cloning Kit (Takara Bio USA Inc.) (S1A Fig). The sequence of the One-STrEP-Flag (OSF) tag was amplified by PCR from a pCAG-OSF plasmid, was generously provided by Dr. Kamitani (Osaka University, Japan), and was introduced at the N-terminus of the C protein of the pCXSN-fWNC plasmid using the In-Fusion HD Cloning Kit (Takara Bio USA Inc.). The region encoding the structural proteins of WNV was cloned into pCR-2.1 (Thermo Fisher Scientific, Waltham, MA, USA), named pCR-CME. Mutations in the sequences of the C protein or structural proteins of WNV were introduced via inverse PCR. The plasmid expressing envelope protein prME (pCXSN-prME) and the plasmid expressing the subgenomic RNA of WNV (pCMV-WNrep-DsRed) were previously constructed [18, 49]. The plasmid expressing the NS proteins (pWNIIrep-GFP, was generously provided by Dr. Robert W. Doms) was previously constructed [50]. Plasmids pCXSN-AMPK and pCXSN-fAMPK were constructed by cloning PCR-amplified AMPK sequence from the total RNA of SK-N-SH cells into the pCXSN or pCXSN-FLAG plasmid, respectively. Plasmid pCXSN-flag-ubiquitin was constructed by cloning PCR-amplified ubiquitin sequence form total RNA of 293T cells into pCXSN plasmid. The lentiviral vector used was CSII-CMV-MCS-IRES2-Bsd, and the packaging plasmids were pCAG-HIVgp and pCMV-VSV-G-Rev, were generously provided by Dr. Miyoshi (Riken, Tsukuba, Japan).

### Antibodies, reagents, and transfection

Rabbit anti-JEV serum that exhibited cross-reactivity with the WNV E protein was produced as previously described [51]. Rabbit anti-NS3 serum was prepared as previously described [49]. The following antibodies were purchased: mouse anti-WNV E protein monoclonal antibody (Merck Millipore, Billerica, MA, USA); mouse anti-ubiquitin monoclonal antibody (Enzo Life Sciences Inc., Farmingdale, NY, USA); mouse anti-actin monoclonal antibody (Wako); mouse anti-LC3 monoclonal antibody, rabbit anti-DDDDK-tag, anti-LC3 and anti-p62 polyclonal antibodies (MBL, Nagoya, Japan); and rabbit anti-AMPK polyclonal and monoclonal antibodies (Cell Signaling Technology, Beverly, MA, USA). MG132, bafilomycin A1, and Tat-Beclin1 peptide were purchased from Calbiochem (San Diego, CA, USA), Wako, and Novus Biologicals (Littleton, CO, USA), respectively.

For peptide treatment, the cells were washed with phosphate-buffered saline (PBS) and treated with the peptide (10 **μ**M) in OPTI-MEM reduced serum media (Thermo Fisher Scientific) acidified with 0.15% (v/v) 6 N HCl. The plasmids were transfected into SK-N-SH cells or SH-SY5Y cells using the Lipofectamine 2000 Transfection Reagent (Thermo Fisher Scientific), Lipofectamine 3000 Transfection Reagent (Thermo Fisher Scientific), or X-tremeGENE HP DNA Transfection Reagent (Roche, Mannheim, Germany) according to the manufacturer’s instructions.

### Recombinant WNV production

The recombinant WNV was produced as previously described [18]. Briefly, the DNA fragments encoding the structural proteins were excised from the pCR-CME plasmid using *Eco*RI. The purified DNA fragment and pCMV-WNrep-DsRed were transfected into HEK-293T cells using Polyethylenimine Max (Polysciences, Warrington, PA, USA). After 3 days, the supernatant was collected and inoculated into Vero cells. Following 5 days, the supernatant of Vero cell cultures was collected and stored at −80°C until use.

### Immunocytochemistry

Cells were fixed with 4% paraformaldehyde for 10 min before washing with PBS. Then, the cells were permeabilized in 0.1% Triton X-100 for 5 min or in 100 **μ**g/ml digitonin for 15 min for the detection of LC3, blocked with 1% bovine serum albumin (BSA)-PBS, and stained overnight at 4°C with the indicated antibodies in 1% BSA-PBS. The immune complexes were visualized following incubation at room temperature for 1 h with Alexa Fluor 488-, Alexa Fluor 555-, Alexa Fluor 594- or Alexa Fluor 647-conjugated secondary antibodies (Thermo Fisher Scientific). The cell nuclei were counterstained with DAPI or Hoechst 33342. Protein aggregates were stained using the PROTEOSTAT Aggresome Detection Kit (Enzo Life Sciences Inc.) according to the manufacturer’s instructions. The dead cells were stained with PI (10 **μ**g/ml). The cells were observed under an IX71 inverted microscope using the DP Manager software (Olympus, Tokyo, Japan) or under LSM 700 or LSM 780 confocal laser scanning microscope with ZEN software (Carl Zeiss, Jena, Germany).

### Immunoblotting

The cells were lysed in 1% Triton buffer (1% Triton X-100; 50 mM Tris–HCl, pH 7.4; 1 mM EDTA; and 0.25 M sucrose). The lysates were centrifuged at 17,500 ×*g* for 15 min at 4°C, to separate supernatants (soluble fraction) and pellets. Pellets were resuspended in 1% SDS in PBS (Triton X-100; insoluble fractions). The cell lysates were fractionated by SDS-PAGE, and the separated proteins were transferred to a PVDF filter (Merck Millipore). The membrane was blocked with 5% skim milk in TBS-T, incubated overnight with each antibody at 4°C, and the immune complexes were detected with HRP-conjugated secondary antibodies and Immobilon Western Chemiluminescent HRP Substrate (Merck Millipore). Chemiluminescence was visualized using the LAS-1000plus Luminescent Image Analyzer system (Fujifilm, Tokyo, Japan) and the ChemiDoc XRS+ Imager (Bio-Rad, Hercules, CA), and the obtained images were analyzed using the Image Lab Software (Bio-Rad).

### Cell viability assay

SK-N-SH cells (20,000 cells) were seeded into 96-well plates and incubated overnight. Then, WNV was inoculated at 1 plaque forming unit (pfu)/cell and incubated for 72 h. Following incubation, the MTT reagent (5 mg/ml; Sigma-Aldrich, St. Louis, MO) was added to each well and the plate was incubated at 37°C for 4 h. After incubation, the culture supernatants were removed and cell lysis solution (2-propanol with 10% Triton-X100 and 0.31% HCl) was added. Absorbance at a wavelength of 560 nm was measured.

### Growth assay and titration

Subconfluent Vero cells or SK-N-SH cells were inoculated with WNV at 0.001 or 1 pfu/cell, respectively, and cultured for 6, 12, 24, or 48 h. The supernatants of WNV-infected cell cultures were collected and stored at −80°C until use for the determination of viral titers. Diluted culture supernatants from the infected cells were inoculated onto monolayers of Vero cells to determine the viral titers. After 1 h incubation at 37°C with rocking, the inoculum was removed, overlay medium (MEM containing 5% FBS and 1.25% methyl cellulose) was added, and the cells were incubated for 4 days. The resultant plaques were visualized by staining with a 0.25% crystal violet solution in 10% formalin.

### Encapsidation and uncoating assay

HEK-293T cells were transfected with pCXSN-fWNC, pCXSN-prME, and pWNIIrep-GFP using Polyethylenimine Max (Polysciences). After 48 h, the supernatant was collected and cell debris was removed by centrifugation. The supernatants were stored at −80°C until use. Total RNA was isolated from the supernatant using Isogen-LS (Nippon Gene, Tokyo, Japan), according to the manufacturer’s protocol, and was reverse-transcribed with random primers using SuperScript III Reverse Transcriptase (Thermo Fisher Scientific). Quantitative PCR was performed with Kapa Probe Fast qPCR kit (Kapa Biosystems, Wilmington, MA, USA) with the 7500 fast Real-Time PCR system (Thermo Fisher Scientific). The WNV primer and probe sequences used were as follows: WNV forward, 5′-GCACGAAGATCTCGATGTCTAAG-3′; WNV reverse, 5′-ATTCCGCGTTTTAGCATATTGAC-3′; and WNV probe, FAM-5′-ACCAGGAGGGCCCGG-3′-MGB.

The amount of VLPs in the each supernatant was standardized by the amount of replicon RNA packaged in each VLP. SH-SY5Y cells were inoculated with the supernatant with the equivalent amount of VLPs for 24 h. The cells were washed twice with PBS and dissociated with 0.25% trypsin. The GFP signal was measured by a FACS Verse system (BD Biosciences, San Diego, CA, USA).

### RT-PCR

Total RNA was isolated using Isogen II (Nippon Gene) according to the manufacturer's protocol and reverse-transcribed with random primers using SuperScript III Reverse Transcriptase (Thermo Fisher Scientific). For detection of mRNA of p62, primer pairs were 5'-CTGCCCAGACTACGACTTGTGT-3' and 5'-TCAACTTCAATGCCCAGAGG-3'.

### Generation of Atg5 knockout SH-SY5Y cells and AMPK knock down 293T cells

Atg5 knockout SH-SY5Y cells were generated by first producing lentiviral vectors via transfection of HEK-293T cells with plasmids encoding Cas9 (pCW-Cas9: Addgene plasmid #50661) [52], pCAG-HIVgp, and pCMV-VSV-G-Rev. Lentiviral vectors were then used to infect SH-SY5Y cells, which were then selected by puromycin to obtain a doxycycline-inducible Cas9 population, and designated as SH-SY5Y Cas9 cells. The sgRNA targeting Atg5 were cloned into the pLX-sgRNA (Addgene plasmid # 50662) [52], and designated as pLX-Atg5-sgRNA. The three sgRNA sequences were the following: ACTTGTTTCACGCTATATC, CTTGTTTCACGCTATATCC, and CAATCGGAAACTCATGGAC. Lentiviruses encoding sgRNAs were produced in HEK-293T cells by co-transfection of pLX-Atg5-sgRNA, pCAG-HIVgp, and pCMV-VSV-G-Rev, and were used to infect the SH-SY5Y Cas9 cells. Following blasticidin selection, Cas9 expression was induced for 4 days using doxycycline. These cells were then cloned by the limiting dilution method. Four clones were picked and further analyzed for Atg5 and LC3 expression by immunoblotting. The oligonucleotide (GTTGCCTACCATCTCATAATAttcaagagaTATTATGAGATGGTAGGCAAC) was subcloned into the pFU6-pGK puro vector, and the resulting plasmid was named pFU6-shAMPK. Lentivirus encoding shAMPK was produced in 293T cells by co-transfection of pFU6-shAMPK and the packaging plasmids, and was used to infect the 293T cells and selected with puromycin for 1 week.

### Protein precipitation

After transfection of pCXSN-flag-ubiquitin, SH-SY5Y cells infected with WNV were washed with PBS and then harvested in 1% Triton buffer. The lysates were immunoprecipitated with 1 μg anti-Flag antibody. Immunoprecipitation was performed by incubating the cell lysates at 4°C for 3 h with 10 μl antibody-coupled Surebeads Protein G Magnetic Beads (Bio-Rad) according to the manufacturer’s protocol. The lysates from SH-SY5Y cells transfected with each plasmid were incubated with Strep-Tactin Sepharose (IBA Lifesciences, Gottingen, Germany) at 4°C for 3 h. The reaction and elution were performed according to the manufacturer’s instructions. The precipitated protein complexes were separated by SDS-PAGE and analysed by immunoblotting.

### Ethics statement

All animal experiments were performed following the basic guidelines for animal experiments of the Ministry of Education, Culture, Sports, Science and Technology (MEXT), Japan. The President of Hokkaido University approved all animal experiments after review by the Institutional Animal Care and Use Committee of Hokkaido University (approval no. 13025).

### WNV inoculation into mice

Six-week-old C57BL/6JJmsSlc mice were obtained from Japan SLC Inc. (Shizuoka, Japan). The animals were anesthetized using sevoflurane and infected with WNV via an intraperitoneal or intracranial inoculation of 100 pfu or 10 pfu per animal, respectively. After euthanasia at 3, 5, or 7 dpi, the brain, spleen, and blood were collected.

### Histology and immunohistochemistry

The collected tissue samples were fixed in 10% phosphate-buffered formalin (pH 7.2) and embedded in paraffin. The sections were stained using Carazzi’s hematoxylin and eosin. For immunohistochemical analysis for the detection of WNV antigen, deparaffinized sections were subjected to antigen retrieval by 0.1% trypsin digestion at room temperature for 10 min. Then, the sections were incubated in 0.3% H_2_O_2_ in methanol (vol/vol) at room temperature for 15 min to quench endogenous peroxidase activity. After blocking with 10% normal goat serum for 10 min, the sections were incubated with primary antibodies at 4°C overnight, followed by 30 min incubation with biotinylated-secondary antibodies that were detected using the Histofine diaminobenzidine Substrate Kit (Nichirei, Tokyo, Japan).

### Quantification and statistical analysis

The immunocytochemical images were recorded with a LSM 700, LSM 780 confocal laser-scanning microscope (Carl Zeiss), or IX71 inverted microscope (Olympus). For each condition of each experiment, at least 3 fields chosen were counted. The number of cells was calculated using Fiji software. Images were processed utilizing the ZEN software (Carl Zeiss) or the DP Manager software (Olympus), and the number of ubiquitin-positive cells, PI-positive cells, LC3 particles, and p62 particles were measured using the Fiji software (http://fiji.sc/Fiji).

Quantification of immunoblotting was performed by densitometry measurements of each lane using Image Lab Software (Bio-Rad), and was normalized to the relative densitometry of the loading control from the same blot.

HE-stained brain sections were recorded with an IX71 inverted microscope (Olympus). Degenerated cells were defined as cells with pyknotic nuclei and eosinophilic cytoplasm. For confocal analysis, pictures were taken on a LSM 780 confocal laser-scanning microscope (Carl Zeiss). For each condition of each experiment, at least 5 fields chosen were counted. The number of cells was calculated using Fiji software.

For panels showing the quantification of image data, at least three fields were counted from a minimum of three biological replicate. Data are presented as the mean ± standard error. Data between two independent groups were analyzed using two-tailed Student’s *t*-test. Data among multiple independent groups were analyzed via one-way ANOVA with Tukey-Kramer test, Scheffe’s F-test, or Dunnett test. The survival curve data were analyzed using the Kaplan–Meier method. The statistical significance was established *a priori* at *p* < 0.05.

## Supporting information

S1 Fig Ubiquitin accumulation in cells expressing mutant C protein of WNV or JEV. (a) Schematic diagram of chimeric C proteins of WNV and JEV. The C protein contains four alpha helixes, and the coding sequences for each helix were replaced between WNV and JEV. Amino acid sequences of the 2^nd^ alpha helix region are shown. (b) SK-N-SH cells were transfected with the indicated plasmids and cultured for 48 h. The cells were double stained for ubiquitin (green) and flag-C (red). Cell nuclei were counterstained with DAPI (blue). Arrowheads indicate ubiquitin signals. Scale bar: 5 **μ**m. (C) Number of ubiquitin-positive cells per transfected cells was counted using the Fiji image software. Data represent the means ± standard error of three independent experiments. Statistical significance was assessed using a one-way ANOVA (*F* = 36.12, *p* < 0.001) followed by Dunnett’s test. **p < 0.01.
(TIF)

S2 Fig Effects of mutations on the properties of WNV. (a) The effects of mutations on infectivity. SK-N-SH cells were inoculated with WNV WT or LA51IT (1 pfu/cell) and E protein-positive cells were counted using flow cytometer at the indicated time point. Data represent the means ± standard error of three independent experiments. (b) The effect of mutations on RNA encapsidation. 293T cells were transfected with plasmids producing virus-like particles (VLPs). The amount of replicon RNA encapsidated in secreted VLPs was analyzed by quantitative RT-PCR. Data represent mean ± standard error of three independent experiments. Statistical significance was assessed using a two-tailed Student’s *t*-test. n. s.: not significant. (c) Effect on mutations on RNA uncoating. SH-SY5Y cells were inoculated with VLPs with WT or LA51IT C protein. After 24 h incubation, the number of GFP-positive cells was analyzed by flow cytometry. Data represent the means ± standard error of three independent experiments. Statistical significance was assessed using a two-tailed Student’s *t*-test. n. s.: not significant.
(TIF)

S3 Fig Analysis of proteasome and autophagy inhibition by WNV infection. (a) The SH-SY5Y cells stably expressing ZsProSensor-1 protein were infected with wild-type (WT) or mutant (LA51IT) WNV (1 pfu/cell) or treated with MG132 (2 **μ**M) as a positive control. After 48 h, the cells were stained for E protein. Scale bar: 100 **μ**m. (b) Autophagosome formation in cells infected with WNV. SK-N-SH cells were infected with WT or LA51IT WNV (1 pfu/cell). After 48 h, the cells were treated with bafilomycin A1 (1 **μ**M) for 6 h and harvested. The harvested cells were stained for LC3 and E protein. Scale bar: 5 **μ**m. (c) Analysis of expression of ATG5 in Atg5 knockout (KO) SH-SY5Y cells. Control or Atg5 KO SH-SY5Y cells were harvested and immunoblot analysis was performed using antibodies against ATG5, LC3, and actin. The positions of the ATG5-ATG12 conjugate and LC3-II are indicated. (d) Examination of the punctate structures of LC3. (Upper) Control or Atg5 KO SH-SY5Y cells were infected with WT or LA51IT WNV (1 pfu/cell). After 48 h, the cells were treated with bafilomycin A1 (1 **μ**M) for 6 h and harvested. The harvested cells were stained for LC3 and E protein. Scale bar: 5 **μ**m. (Lower) Numbers of LC3 particles per WNV-infected cells were counted using Fiji image software. Data represent the means ± standard error of four independent experiments. Statistical significance was assessed using a one-way ANOVA (*F* = 12.31, *p* < 0.001) followed by the Tukey-Kramer test. *p < 0.05. (e) mRNA expression of p62 in cells infected with WNV. The cells infected with WNV were harvested at 48 hpi and analyzed by RT-PCR to detect p62, viral genome, and actin levels.
(TIF)

S4 Fig Effect of autophagy induction on the accumulation and cell death. (a) Schematic diagram of autophagy induction signaling. (b) SK-N-SH cells were transfected with the plasmid expressing C protein. After 48 h, the cells were treated with Tat-Beclin1 for 3 h and harvested. The harvested cells were stained for viral antigen (red) and ubiquitin (green, upper) or LC3 (green, lower). Nuclei were stained with DAPI. Arrowheads indicate ubiquitin signal (upper) or LC3 signal (lower). Scale bars: 5 **μ**m. (c) SH-SY5Y cells were infected with WNV (1 pfu/cell). After 48 h, the cells were treated with Tat-Beclin1 (2 **μ**M) for 24 h, and dead cells were stained with PI. PI-positive cells were counted using the Fiji image software. Data represent the means ± standard error of three independent experiments. Statistical significance was assessed using a two-tailed Student’s *t*-test. **p < 0.01. (d) SH-SY5Y Cas9 cells (control) or SH-SY5Y Atg5 KO cells (Atg5 KO) were infected with WT or LA51IT WNV (1 pfu/cell). The cells were harvested at 48 hpi, and the harvested lysates were separated into a Triton X-100-soluble fraction for the detection of Atg5, NS3, and actin and a Triton X-100-insoluble fraction, for detection of ubiquitin, by immunoblotting.
(TIF)

S5 Fig Effect of C protein on AMPK expression. (a) SK-N-SH cells were transfected with a plasmid expressing WT, LA51IT, or JEV C protein. After 72 h, cells were treated with MG132 (2 **μ**M) for 3 h and analyzed by immunoblotting using anti-AMPK, -flag, and -actin antibodies. (b) SK-N-SH cells were transfected with a plasmid expressing NS4A or all of NS proteins. After 72 h, cells were analyzed by immunoblotting using anti-AMPK, -flag, -NS3, and -actin antibodies. (c) SH-SY5Y cells were transfected with plasmids expressing flag-ubiquitin and cultured for 24 h. The cells were infected with WNV WT or LA51IT (1 pfu/cell). After 48 h, the cells were treated with DMSO for 3 h before anti-Flag immunoprecipitation (IP) and immunoblotting were performed. (d) Analysis of expression of ATG5 in Atg5 knockout (KO) SH-SY5Y cells. Control or AMPK knockdown (KD) SH-SY5Y cells were harvested and immunoblot analysis was performed using antibodies anti-AMPK and -actin antibodies. (e) SH-SY5Y cells were co-transfected with plasmids expressing AMPK and WT, LA51IT, or JE C protein with One-STrEP-Flag (OSF) tag and cultured for 48 h. The cells were treated with DMSO for 3 h before precipitation by Strep-Tactin. The OSF-C protein complex was analyzed by immunoblotting.
(TIF)

S6 Fig Model for the pathogenesis of West Nile encephalitis by C protein. In the cells infected with West Nile virus, C protein facilitates AMPK ubiquitination and proteasome-mediated degradation. This degradation inhibits autophagy that constitutively eliminates protein aggregates under normal conditions. The inhibition of autophagy induces the accumulation of protein aggregates, resulting in cell death and neurological disease.
(TIF)

S7 Fig Protein sequence alignment of C protein from Flaviviruses. Protein sequences from West Nile virus (WNV) NY99 strain (accession number: AB185914), WNV Eg101 strain (AF260968), WNV FCG strain (M12294), WNV B956 strain (AY532665), Kunjin virus (KUNV) MRM61C strain (D00246), Japanese encephalitis virus (JEV) JaGAr 01 strain (AF069076), JEV Beijing-1 strain (L48961), JEV Sw/Mie/34/2004 strain (AB698909), JEV FJ03-39 strain (JN381859), JEV B-1381-85 strain (GQ902061), JEV Muar strain (HM596272), JEV XZ0934 strain (JF915894), Zika virus (ZIKV) PRVABC59 strain (KU501215), Dengue virus (DENV) D/Hu/India/NIID74/2009 strain (LC367234), and Tick-borne encephalitis virus (TBEV) Oshima 5–10 strain (MF374487) were aligned using the Clustal W program. Each alpha-helix region is boxed within dotted line. The region shaded in yellow indicates the amino acid at position 51 and 52.
(TIF)
