# Supplementary figures and images for: West Nile virus capsid protein inhibits autophagy by AMP-activated protein kinase degradation in neurological disease development
Source: PLoS Pathog. 2020 Jan 23;16(1):e1008238. doi: 10.1371/journal.ppat.1008238 (PMC6977728; doi:10.1371/journal.ppat.1008238)

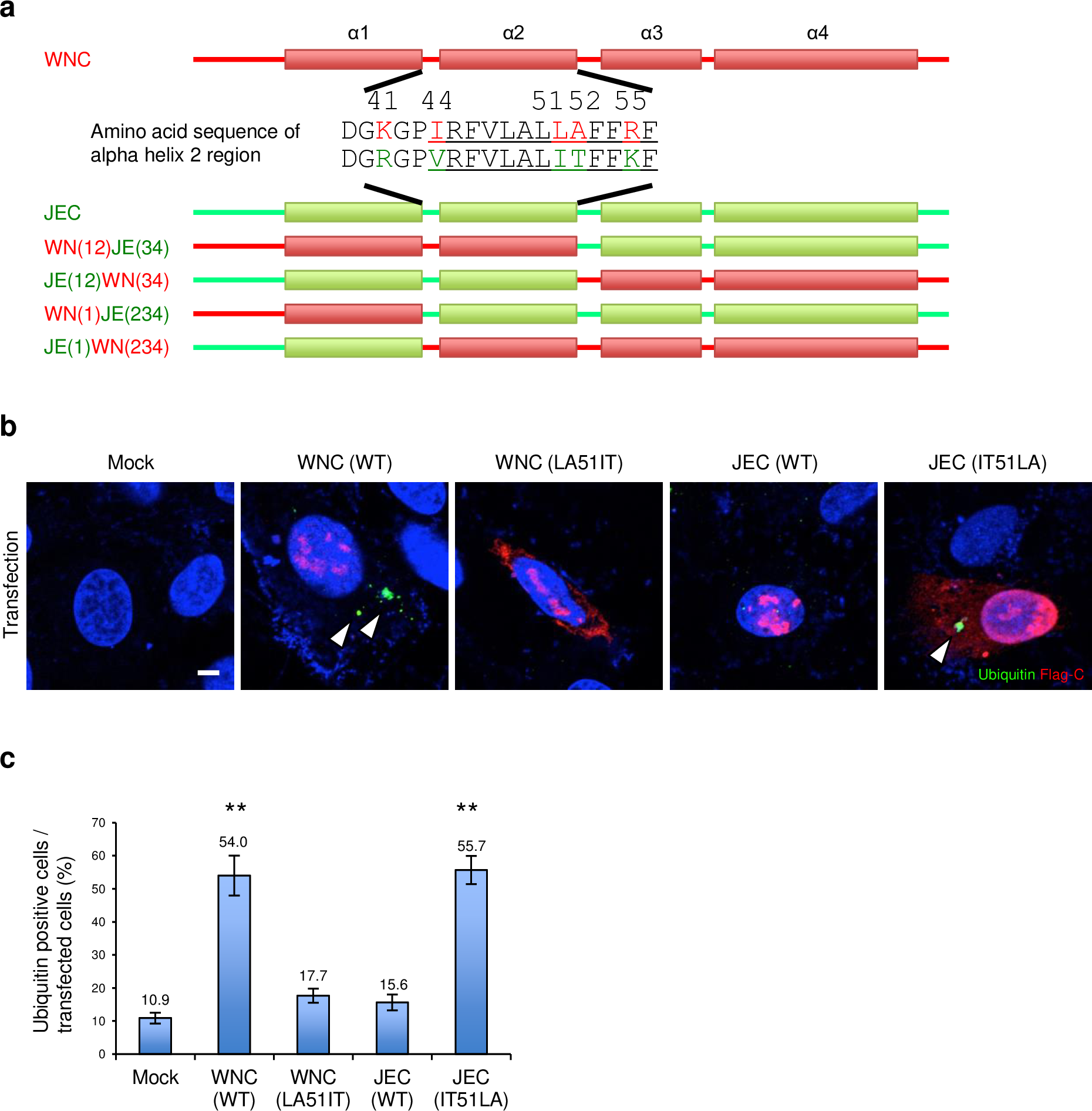

Supplement: S1 Fig — (a) Schematic diagram of chimeric C proteins of WNV and JEV. The C protein contains four alpha helixes, and the coding sequences for each helix were replaced between WNV and JEV. Amino acid sequences of the 2nd alpha helix region are shown. (b) SK-N-SH cells were transfected with the indicated plasmids and cultured for 48 h. The cells were double stained for ubiquitin (green) and flag-C (red). Cell nuclei were counterstained with DAPI (blue). Arrowheads indicate ubiquitin signals. Scale bar: 5 μm. (C) Number of ubiquitin-positive cells per transfected cells was counted using the Fiji image software. Data represent the means ± standard error of three independent experiments. Statistical significance was assessed using a one-way ANOVA (F = 36.12, p < 0.001) followed by Dunnett’s test. **p < 0.01. (TIF) [file ppat.1008238.s001.tif]

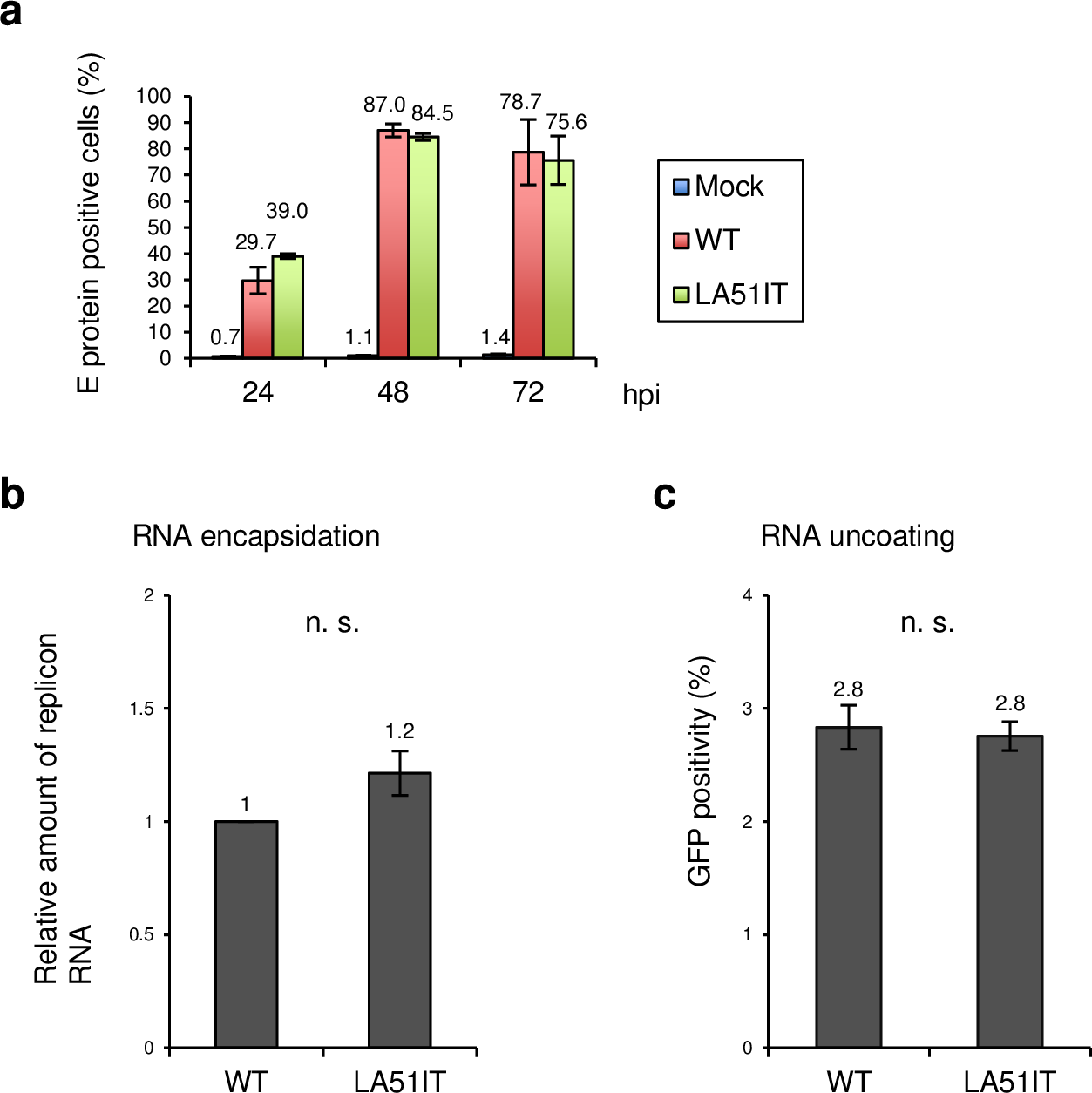

Supplement: S2 Fig — (a) The effects of mutations on infectivity. SK-N-SH cells were inoculated with WNV WT or LA51IT (1 pfu/cell) and E protein-positive cells were counted using flow cytometer at the indicated time point. Data represent the means ± standard error of three independent experiments. (b) The effect of mutations on RNA encapsidation. 293T cells were transfected with plasmids producing virus-like particles (VLPs). The amount of replicon RNA encapsidated in secreted VLPs was analyzed by quantitative RT-PCR. Data represent mean ± standard error of three independent experiments. Statistical significance was assessed using a two-tailed Student’s t-test. n. s.: not significant. (c) Effect on mutations on RNA uncoating. SH-SY5Y cells were inoculated with VLPs with WT or LA51IT C protein. After 24 h incubation, the number of GFP-positive cells was analyzed by flow cytometry. Data represent the means ± standard error of three independent experiments. Statistical significance was assessed using a two-tailed Student’s t-test. n. s.: not significant. (TIF) [file ppat.1008238.s002.tif]

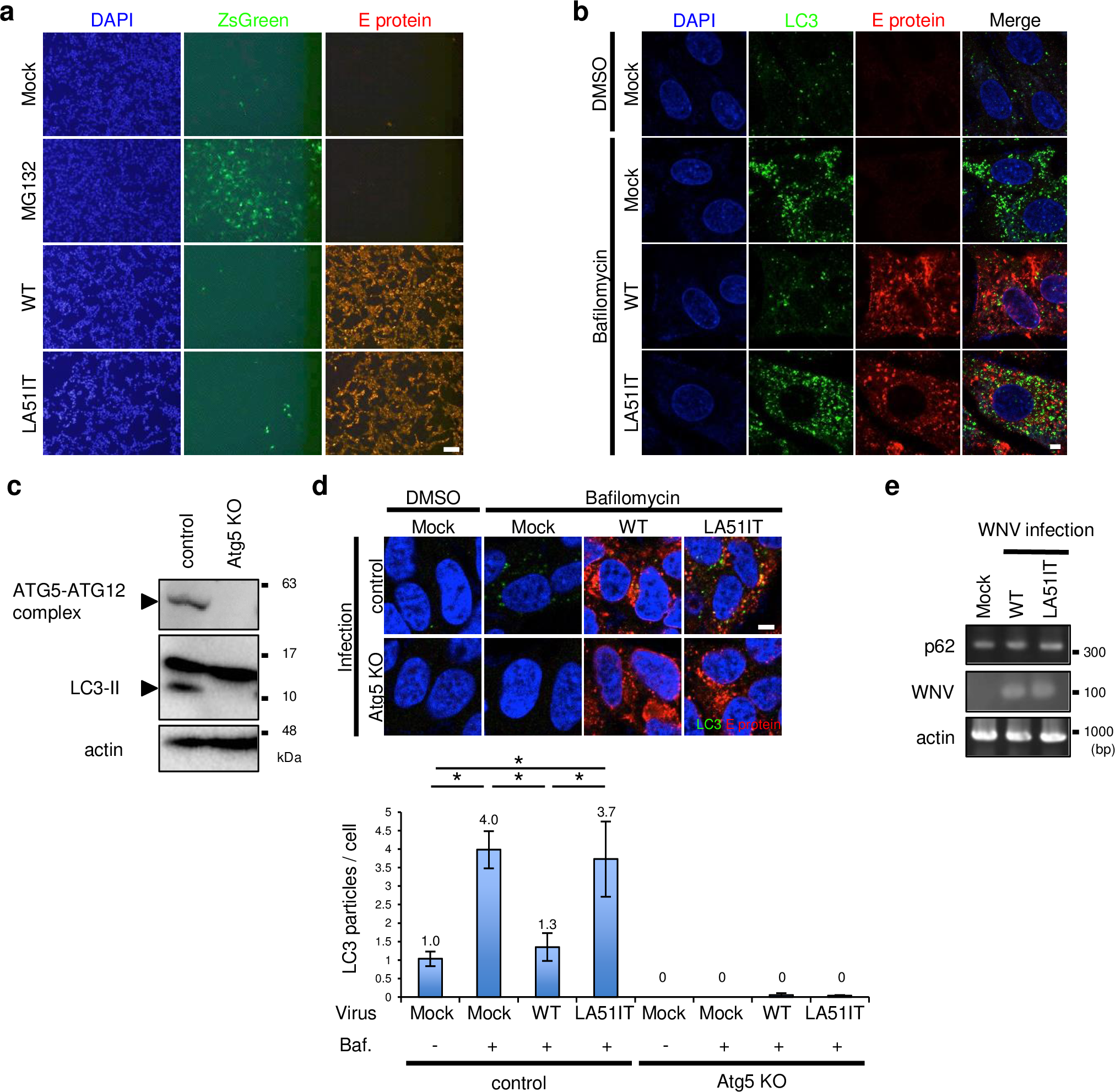

Supplement: S3 Fig — (a) The SH-SY5Y cells stably expressing ZsProSensor-1 protein were infected with wild-type (WT) or mutant (LA51IT) WNV (1 pfu/cell) or treated with MG132 (2 μM) as a positive control. After 48 h, the cells were stained for E protein. Scale bar: 100 μm. (b) Autophagosome formation in cells infected with WNV. SK-N-SH cells were infected with WT or LA51IT WNV (1 pfu/cell). After 48 h, the cells were treated with bafilomycin A1 (1 μM) for 6 h and harvested. The harvested cells were stained for LC3 and E protein. Scale bar: 5 μm. (c) Analysis of expression of ATG5 in Atg5 knockout (KO) SH-SY5Y cells. Control or Atg5 KO SH-SY5Y cells were harvested and immunoblot analysis was performed using antibodies against ATG5, LC3, and actin. The positions of the ATG5-ATG12 conjugate and LC3-II are indicated. (d) Examination of the punctate structures of LC3. (Upper) Control or Atg5 KO SH-SY5Y cells were infected with WT or LA51IT WNV (1 pfu/cell). After 48 h, the cells were treated with bafilomycin A1 (1 μM) for 6 h and harvested. The harvested cells were stained for LC3 and E protein. Scale bar: 5 μm. (Lower) Numbers of LC3 particles per WNV-infected cells were counted using Fiji image software. Data represent the means ± standard error of four independent experiments. Statistical significance was assessed using a one-way ANOVA (F = 12.31, p < 0.001) followed by the Tukey-Kramer test. *p < 0.05. (e) mRNA expression of p62 in cells infected with WNV. The cells infected with WNV were harvested at 48 hpi and analyzed by RT-PCR to detect p62, viral genome, and actin levels. (TIF) [file ppat.1008238.s003.tif]

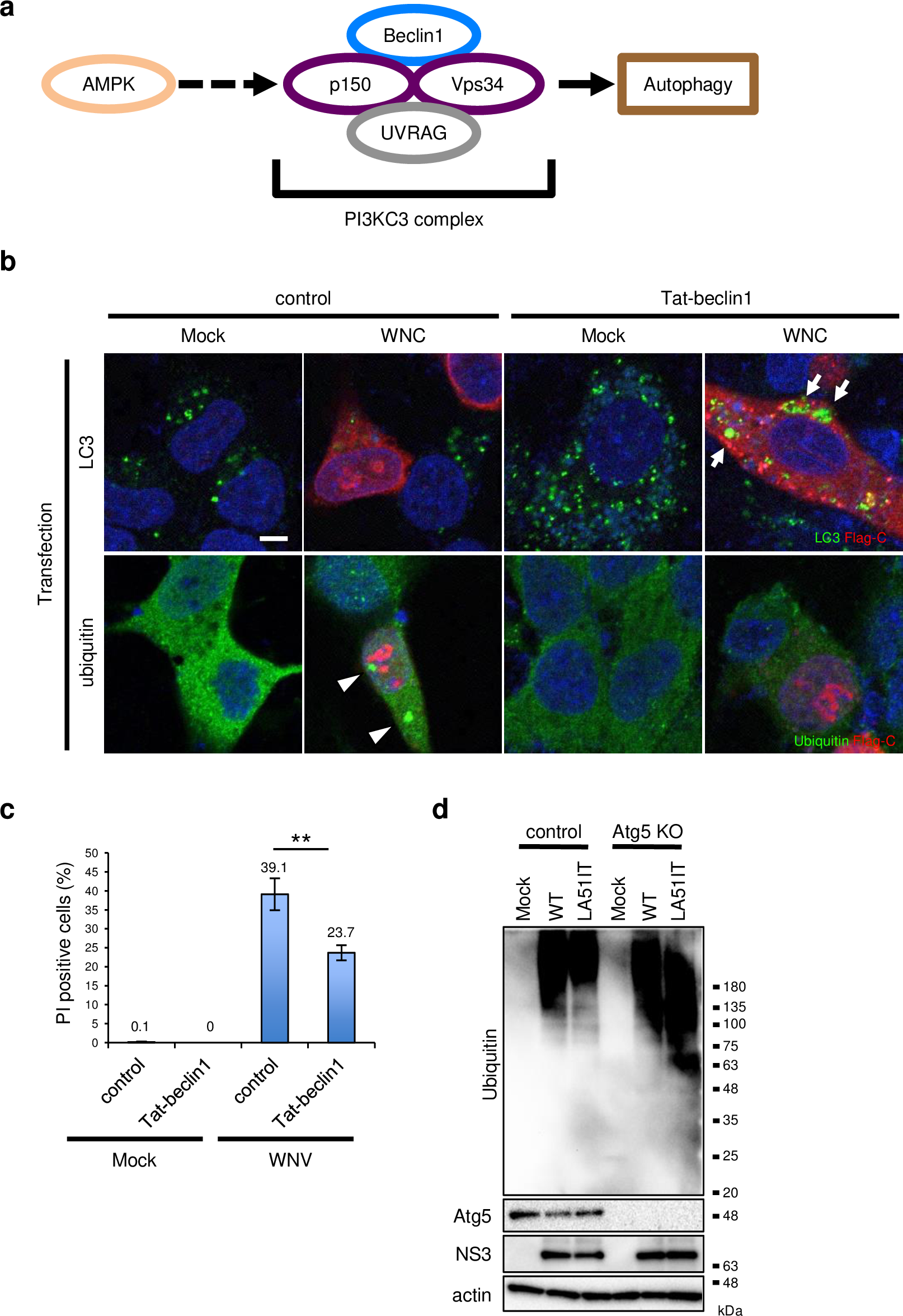

Supplement: S4 Fig — (a) Schematic diagram of autophagy induction signaling. (b) SK-N-SH cells were transfected with the plasmid expressing C protein. After 48 h, the cells were treated with Tat-Beclin1 for 3 h and harvested. The harvested cells were stained for viral antigen (red) and ubiquitin (green, upper) or LC3 (green, lower). Nuclei were stained with DAPI. Arrowheads indicate ubiquitin signal (upper) or LC3 signal (lower). Scale bars: 5 μm. (c) SH-SY5Y cells were infected with WNV (1 pfu/cell). After 48 h, the cells were treated with Tat-Beclin1 (2 μM) for 24 h, and dead cells were stained with PI. PI-positive cells were counted using the Fiji image software. Data represent the means ± standard error of three independent experiments. Statistical significance was assessed using a two-tailed Student’s t-test. **p < 0.01. (d) SH-SY5Y Cas9 cells (control) or SH-SY5Y Atg5 KO cells (Atg5 KO) were infected with WT or LA51IT WNV (1 pfu/cell). The cells were harvested at 48 hpi, and the harvested lysates were separated into a Triton X-100-soluble fraction for the detection of Atg5, NS3, and actin and a Triton X-100-insoluble fraction, for detection of ubiquitin, by immunoblotting. (TIF) [file ppat.1008238.s004.tif]

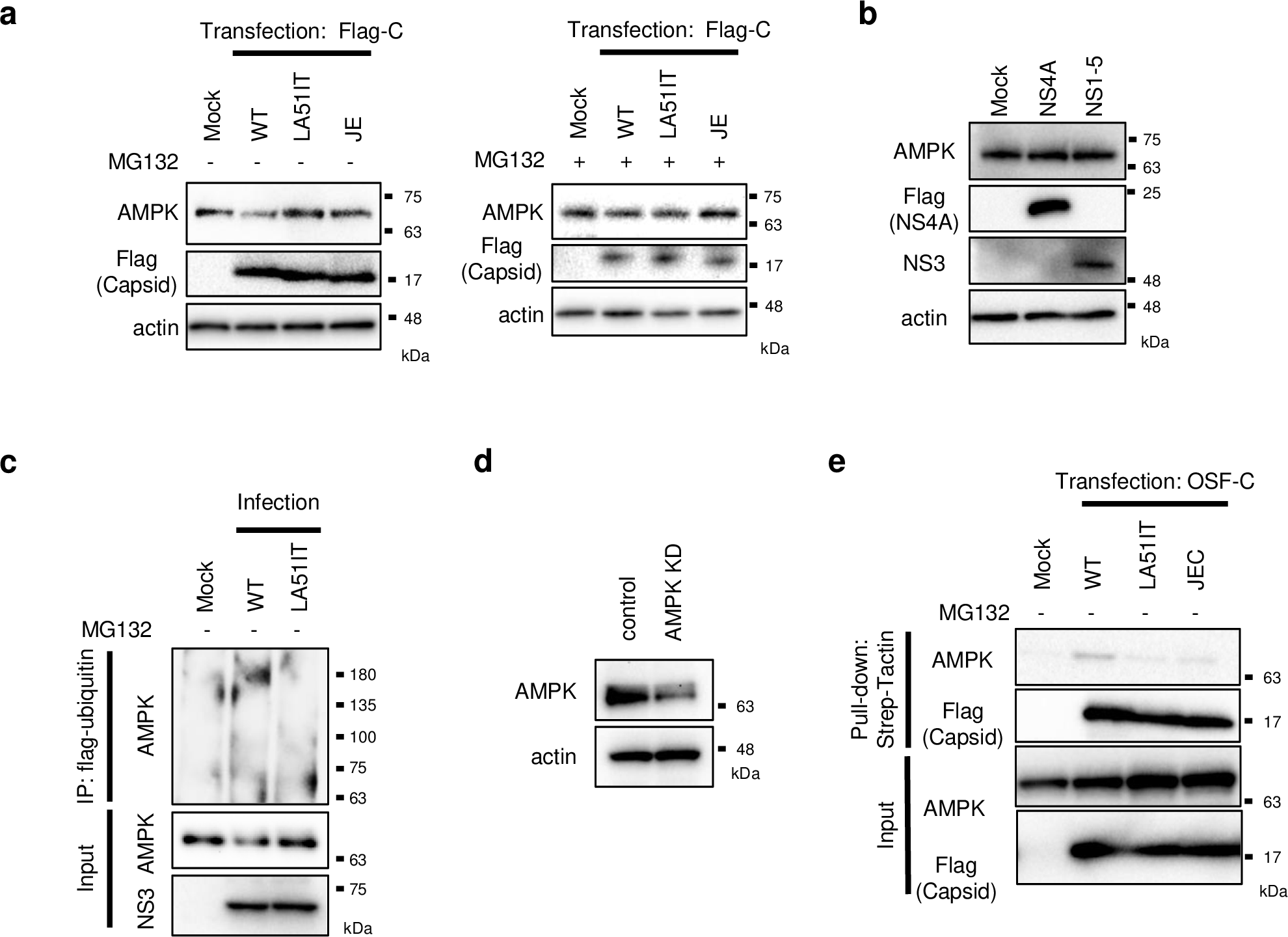

Supplement: S5 Fig — (a) SK-N-SH cells were transfected with a plasmid expressing WT, LA51IT, or JEV C protein. After 72 h, cells were treated with MG132 (2 μM) for 3 h and analyzed by immunoblotting using anti-AMPK, -flag, and -actin antibodies. (b) SK-N-SH cells were transfected with a plasmid expressing NS4A or all of NS proteins. After 72 h, cells were analyzed by immunoblotting using anti-AMPK, -flag, -NS3, and -actin antibodies. (c) SH-SY5Y cells were transfected with plasmids expressing flag-ubiquitin and cultured for 24 h. The cells were infected with WNV WT or LA51IT (1 pfu/cell). After 48 h, the cells were treated with DMSO for 3 h before anti-Flag immunoprecipitation (IP) and immunoblotting were performed. (d) Analysis of expression of ATG5 in Atg5 knockout (KO) SH-SY5Y cells. Control or AMPK knockdown (KD) SH-SY5Y cells were harvested and immunoblot analysis was performed using antibodies anti-AMPK and -actin antibodies. (e) SH-SY5Y cells were co-transfected with plasmids expressing AMPK and WT, LA51IT, or JE C protein with One-STrEP-Flag (OSF) tag and cultured for 48 h. The cells were treated with DMSO for 3 h before precipitation by Strep-Tactin. The OSF-C protein complex was analyzed by immunoblotting. (TIF) [file ppat.1008238.s005.tif]

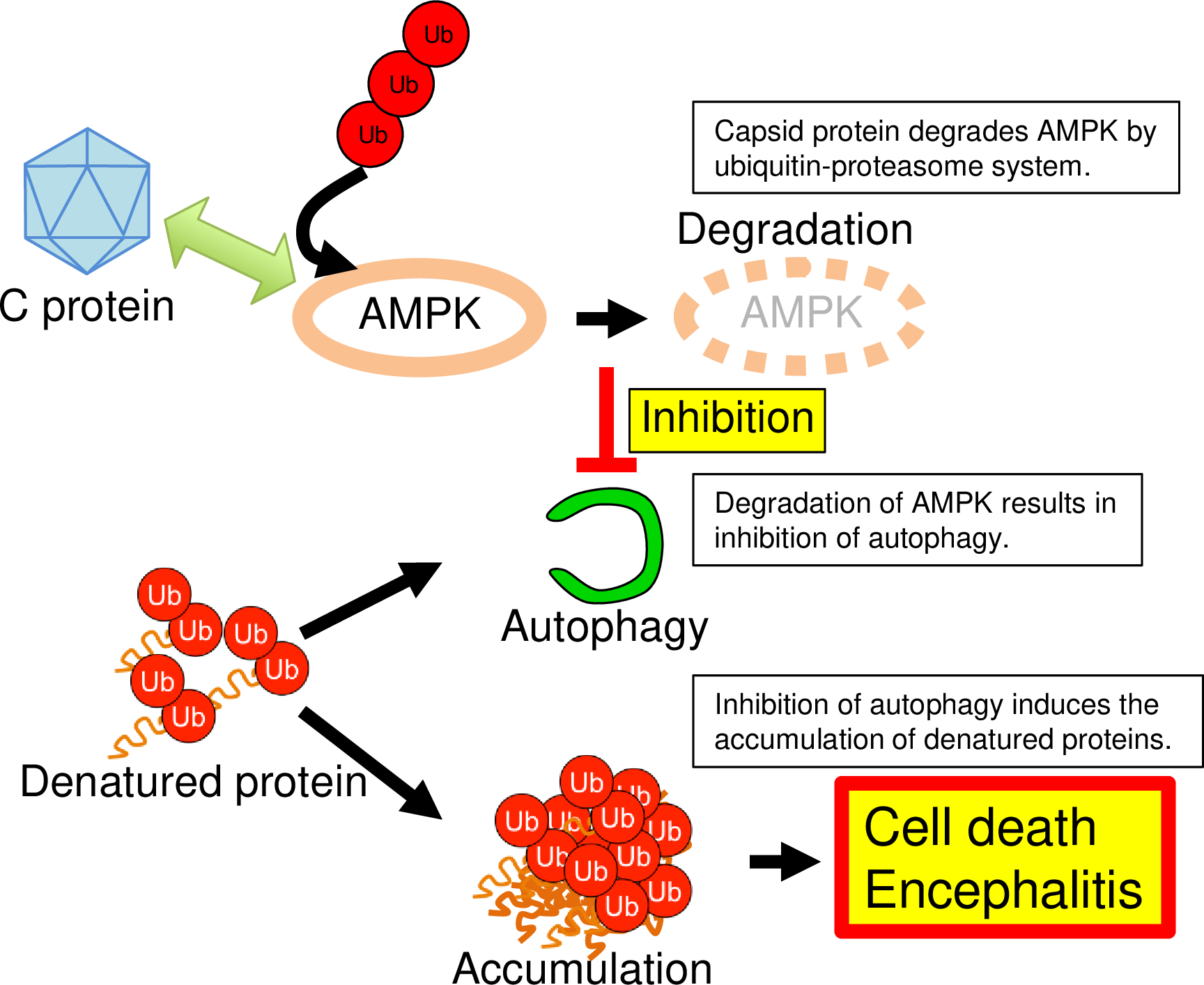

Supplement: S6 Fig — In the cells infected with West Nile virus, C protein facilitates AMPK ubiquitination and proteasome-mediated degradation. This degradation inhibits autophagy that constitutively eliminates protein aggregates under normal conditions. The inhibition of autophagy induces the accumulation of protein aggregates, resulting in cell death and neurological disease. (TIF) [file ppat.1008238.s006.tif]

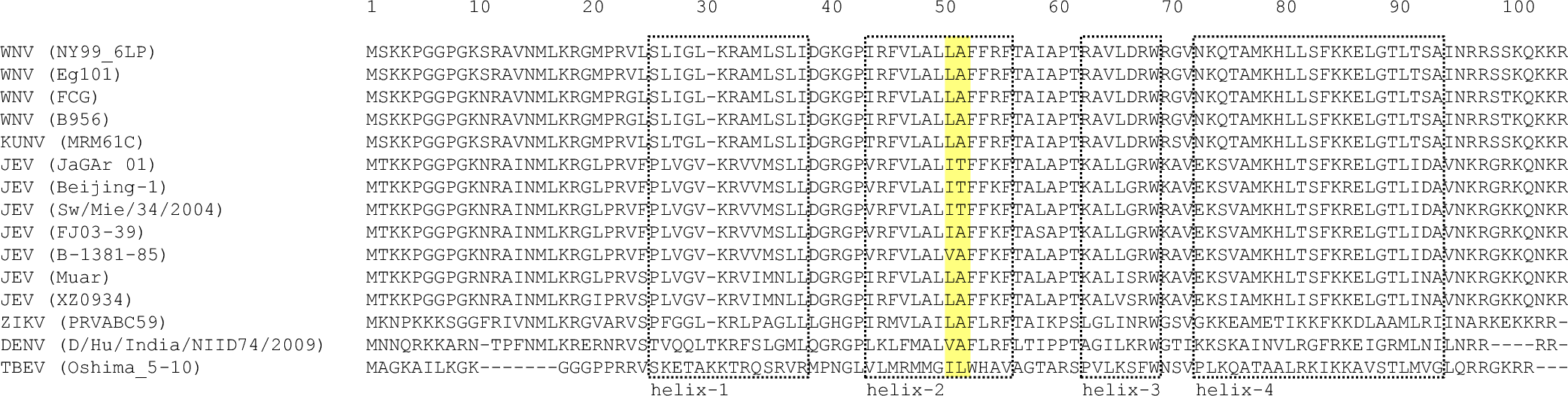

Supplement: S7 Fig — Protein sequences from West Nile virus (WNV) NY99 strain (accession number: AB185914), WNV Eg101 strain (AF260968), WNV FCG strain (M12294), WNV B956 strain (AY532665), Kunjin virus (KUNV) MRM61C strain (D00246), Japanese encephalitis virus (JEV) JaGAr 01 strain (AF069076), JEV Beijing-1 strain (L48961), JEV Sw/Mie/34/2004 strain (AB698909), JEV FJ03-39 strain (JN381859), JEV B-1381-85 strain (GQ902061), JEV Muar strain (HM596272), JEV XZ0934 strain (JF915894), Zika virus (ZIKV) PRVABC59 strain (KU501215), Dengue virus (DENV) D/Hu/India/NIID74/2009 strain (LC367234), and Tick-borne encephalitis virus (TBEV) Oshima 5–10 strain (MF374487) were aligned using the Clustal W program. Each alpha-helix region is boxed within dotted line. The region shaded in yellow indicates the amino acid at position 51 and 52. (TIF) [file ppat.1008238.s007.tif]
